# Magnetized Micropillar-Enabled Wearable Sensors for Touchless and Intelligent Information Communication

**DOI:** 10.1007/s40820-021-00720-5

**Published:** 2021-09-14

**Authors:** Qian Zhou, Bing Ji, Fengming Hu, Jianyi Luo, Bingpu Zhou

**Affiliations:** 1 Joint Key Laboratory of the Ministry of Education, Institute of Applied Physics and Materials Engineering, University of Macau, Avenida da Universidade, Taipa, Macau, 999078 P. R. China; 2 School of Applied Physics and Materials, Research Center of Flexible Sensing Materials and Devices, Wuyi University, Jiangmen, 529020 P. R. China

**Keywords:** Electronic skin, Human–machine interaction, Cryptic information communication, Magnetic field sensing, Tilted magnetized micropillar

## Abstract

The wearable sensors have recently attracted considerable attentions as communication interfaces through the information perception, decoding, and conveying process. However, it is still challenging to obtain a sensor that can convert detectable signals into multiple outputs for convenient, efficient, cryptic, and high-capacity information transmission. Herein, we present a capacitive sensor of magnetic field based on a tilted flexible micromagnet array (t-FMA) as the proposed interaction interface. With the bidirectional bending capability of t-FMA actuated by magnetic torque, the sensor can recognize both the magnitude and orientation of magnetic field in real time with non-overlapping capacitance signals. The optimized sensor exhibits the high sensitivity of over 1.3 T^−1^ and detection limit down to 1 mT with excellent durability. As a proof of concept, the sensor has been successfully demonstrated for convenient, efficient, and programmable interaction systems, e.g., touchless Morse code and Braille communication. The distinguishable recognition of the magnetic field orientation and magnitude further enables the sensor unit as a high-capacity transmitter for cryptic information interaction (e.g., encoded ID recognition) and multi-control instruction outputting. We believe that the proposed magnetic field sensor can open up a potential avenue for future applications including information communication, virtual reality device, and interactive robotics.

**Supplementary Information:**

The online version contains supplementary material available at 10.1007/s40820-021-00720-5.

## Highlights

- A wearable capacitive sensor, which can recognize both the magnitude and orientation of magnetic field with non-overlapping capacitance signals, was proposed as touchless and intelligent communication channel.
- The integrated sensor exhibited the high sensitivity of over 1.3 T^-1^ and detection limit down to 1 mT with excellent durability (over 10,000 cycles).
- The sensor revealed as an efficient and ternary interface with high-capacity for information interaction, e.g., Morse code, Braille communication, and multi-control instruction.

**Supplementary Information:**

The online version contains supplementary material available at 10.1007/s40820-021-00720-5.

## Graphical abstract

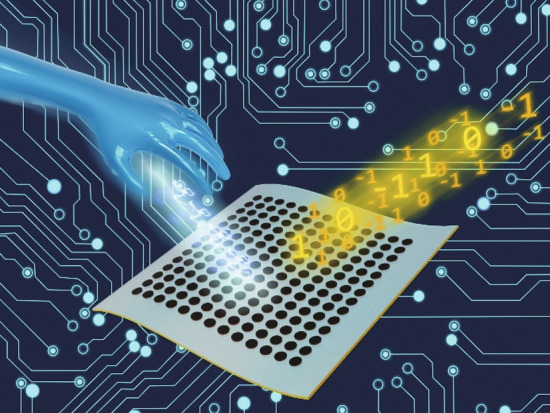

## Introduction

Human–machine interaction (HMI) has been considered as one of the mainstreams in the future with tremendous potentials in Internet of things (IoT), soft robotics, and virtual reality (VR) system, etc. [1–3]. The key component of an HMI or intelligent system is the communication interface that can swiftly transmit commands from humans to machines, or provide timely and precise feedback conversely [4–6]. Particularly, recent advances in flexible and smart sensors, which can directly convert external stimuli into detectable electric signals, have revealed the suitability as communication devices for information capturing, encoding and transferring [7–11]. With such wearable sensors, the encoded electric commands can be produced via customized external stimuli and conveniently transmitted to the target machines for further operations. Furthermore, the feedback or carried information can also be decoded by the devices that mimics the sensing capability of human being [12–14]. The HMI system thus builds a close loop which not only enables the command delivery for intelligent target controls, but also ensures the individuals to be well informed by the feedback for further decision making.

Facing with the emerging age of information explosion, it is of great significance to implement reliable sensor devices that can transmit multiple information via a single unit for the efficient and convenient HMI system. Developing the high-performance sensor devices that can generate multi-level electric signals under different intensities of external stimuli has been demonstrated as an effective approach to improve the capacity of HMI system for information conversion/transmission [15, 16]. Such an approach, however, requires the sophisticated design to simultaneously realize the high sensitivity with high signal-to-noise ratio and the excellent linear sensing capability of the sensor to provide distinguishable electric signals. Moreover, the imprecise input of stimulus intensity caused by individual differences may lead to the inaccurate information conversion/transmission. Integrating sensor units into an array assisted by turning on/off the external stimuli onto each unit to transmit the information can avoid the high dependence on the sensing performance of sensors and the influence of individual differences of users [17–19]. Nevertheless, the limited signal output within a single unit imposes the high demand on the amounts of integrated units to meet the needs of massive information communications, which somehow increases the difficulty of data processing/conversion and reduces the portability. Although developing multifunctional sensors that can respond to different stimuli (e.g., pressure and strain) with non-overlapping electric signals may address the problems mentioned above, the exertion of different kinds of stimulus somehow imposes the inconvenience especially when transmitting a large amount of complex information [20–22]. Therefore, it is still challenging to develop a sensor unit that can conveniently, simply, and reliably output multiple signals under same kind of stimulus to realize the massive information communication of HMI devices for overall system simplification.

Herein, we presented a capacitive sensor of magnetic field based on cilia-like tilted flexible micromagnet array (t-FMA) fabricated with magnetized NdFeB/PDMS micro-pillars for touchless, high-efficient, convenient, and programmable information communications in HMI. Actuated by the magnetic torque, the t-FMA could be bidirectionally deflected under different orientations (i.e., North/South poles) of external magnetic field, which induced the capacitance variation of the sensor correspondingly. Therefore, the sensor could not only perceive the magnitude of magnetic field with different capacitance intensities, but also recognized the orientation of magnetic field with non-overlapping signals (i.e., positive and negative capacitance). The optimized sensor exhibited the high sensitivity over 1.3 T^−1^, low detection limit down to 1 mT, and excellent durability under each pole of magnetic field, which could be potentially used for the real detection of magnetic field. Moreover, by inputting the magnetic field with combinatorial magnitude and orientation, the sensor could output multiple signals under same kind of stimulus in a touchless way for information communication in HMI system. Specifically, the North pole, South pole, and absence of external magnetic field could be encoded as ‘ + 1’, ‘-1’, and ‘0’ to conveniently form a ternary coding system, which enabled the magnetic field as a high capacity and cryptic information carrier. The magnetic field sensor could then serve as a flexible and wearable transmitter to either decode/recognize or actively output the desired information for touchless, efficient, and convenient communication. We experimentally demonstrated that the sensor unit could serve as a convenient platform for the efficient and touchless Morse code output by simply switching the North/South poles of the magnetic field. The programmable Braille interaction system without abundant prefixes was also successfully demonstrated assisted by the integrated sensor units and electromagnet array. Moreover, utilizing the invisibility and ternary coding system of magnetic field, a large amount of cryptic information such as hidden patterns and encoded identity information (ID) could be encoded in the magnetic fields of a small-scale integrated flexible magnet array. The sensor array could then serve as a decoder to identify the cryptic information without the high demand on numerous sensor units taking advantages of the recognition of both the magnitude and orientation of magnetic field. The magnetic field with ternary coding system as stimulus input also enabled the sensor as a high-capacity transmitter to output multi-bit coded control instructions without direct contact. These results experimentally proved the profound significance of the proposed sensor as the intelligent interfaces or communication channels for potential applications in human–machine interaction device, soft robotics, and virtual reality device, etc.

## Experimental Section

### Fabrication of Wearable Capacitive Sensor Based on Cilia-like t-FMA Dielectric

The neodymium–iron–boron microparticles ((NdFeB, MQFP B +) Magnequench, Tianjin, China) were firstly mixed with the polydimethylsiloxane (gel of PDMS (Sylgard 184, Dow Corning, USA) and curing agent with a typical mass ratio of 10:1) to obtain the NdFeB/PDMS mixture. The NdFeB/PDMS mixture with certain NdFeB content was then poured onto the as-prepared template with conversed tilted micro-pillar array and degassed for 5 min to ensure the full filling of the mixture in the template, followed by a spin-coating process (500 rpm, 10 s) to obtain a smooth film (detailed fabrication of the template can be referred to Fig. S1). After placing a copper fiber electrode on the patterned area, the product was moved to the oven (80 °C) for 4 h. The PDMS gel was then spin-coated to the product (700 rpm, 20 s) and cured (80 ℃ for 4 h) to form an encapsulation layer. After peeling off from the template and employing the magnetization process, the t-FMA dielectric was obtained. We herein selected the ferrimagnetic NdFeB as magnetic materials because of its high energy product, large coercive field, and high remanent induction. Moreover, the isotropic NdFeB is also inexpensive because of the comparatively large abundance of Nd and Fe, and it has good thermal aging characteristics [24, 25]. With these features, the employed NdFeB conduces to the realization of high-performance, stable sensors via a relatively facile and cost-effective way. Attributed to the micro-engraving technology, the dimensions of the t-FMA (e.g., diameter, density, height, and tilted angle) could be flexibly regulated by altering the template. To simplify the investigation, the dimension parameters including diameter (300 μm), density (edge distance of adjacent pillars of 1.7 mm), vertical height (~ 1.4 mm) of t-FMA were fixed in this work. The detailed discussion regarding the decision of these dimensional parameters will be provided in the following sections. To assemble the sensor, the t-FMA was dipped into an uncured PDMS film with immersion depth of ~ 200 μm for 30 s, and was then moved to combine with another copper fiber electrode encapsulated by PDMS layer. After the solidification (50 °C for 4 h), the assembled capacitive sensor of magnetic field was obtained. The prepared products possessed the fixed area of 1 $\times$ 1 cm^2^ unless otherwise specialized.

### Preparation of Flexible Magnet Array

The mixture of NdFeB/PDMS (75 wt% of NdFeB) was poured onto a reusable template with rectangular groove array, which was obtained by micro-engraving technology as mentioned above. Each rectangle covers the area of 5 × 5 mm^2^, depth of 0.5 mm, and edge distance of 5 mm. After degassed for 20 min, the mixture over the patterns was removed off from the template surface with a plastic strip, followed by the solidification under 80 ℃ for 4 h. The PDMS gel was then spin-coated (700 rpm for 20 s) to the product to form a smooth film. After solidification (80 ℃, 4 h), the PDMS film with square-structured NdFeB/PDMS was peeled off from the template. The entire product was then magnetized in an out-of-plane magnetizing field (~ 3 T) to obtain the flexible magnet array.

### Measurements and Characterizations

The spin-coating process was carried out by the KW-4A spin-coater (Institute of Microelectronics, Chinese Academy of Sciences, Beijing, China). The SEM and EDX images were obtained by the scanning electron microscopy (Sigma FE-SEM, Zeiss Corporation, Germany). The optical images were captured by the optical microscopy purchased from Carl Zeiss Microscopy (Jena, Germany) and Olympus America (Center Valley, PA). The intensity and direction of magnetic field were evaluated via the commercial gauss meter (Model HT20, HengTong Magnetoelectricity Ltd., Shanghai, China). The stimulation of magnetic field was accurately controlled by fixing the permanent NdFeB magnet to a micro-displacement platform (MAR 100–90, Zolix Instruments Co. Ltd., China) with a motor (MC600, Zolix Instruments Co. Ltd., China). The electromagnetic field was controlled by the electromagnet assisted with a power supply (0–32 V; ELECALL electric Co., Ltd., Yueqing, China). The mechanical properties of the NdFeB/PDMS are carried out at room temperature (25 °C) by MTS (model E44, EXCEED) testing machine. The hysteresis loops were measured by a PPMS DynaCool instrument using the vibrating sample magnetometry at room temperature. The loading/unloading of normal pressures were precisely controlled by the same maneuvering platform and measured by an electronic balance (Sartorius Lab Instruments GmbH & Co.KG, 37,070 Gottingen, Germany). The real-time capacitance signals were obtained and recorded by Keysight E4980AL under a typical voltage of 1 V and frequency of 10 kHz unless otherwise specialized. The wearable demonstrations were carried out by a research volunteer with ensured safety during the characterization. Informed consents were obtained from the volunteer for the related tests.

## Results and Discussion

### Principle of Touchless Intelligent Communication System

**Fig. 1:**
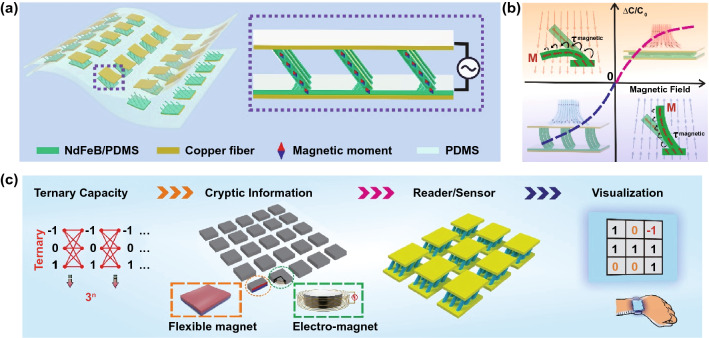
**a **Structure and magnetic property design of the flexible magnetic field sensor based on t-FMA. **b** Working mechanism of the flexible magnetic field sensor. ***M*** and *τ*^*magnetic*^ represent the magnetic moment vector and magnetic torque, respectively. **c** Schematic of encoding, decoding and extraction of the targeted information based on our proposed touchless and cryptic information recognition system

The magnetic field, with unique properties such as invisibility, orientation, and spatial distribution, can serve as an effective medium for cryptic, efficient, and touchless information communication. To fully explore such capabilities, the sensors should not only respond to the magnitude of magnetic field, but also be capable to provide different feedbacks regarding the orientation of the magnetic field, e.g., the North/South pole. Figure 1a, b depicts the structure and working mechanism of our proposed HMI device with such functions. The device consists of a series of sensor unit, and each unit is indeed an integrative capacitor composed of the magnetized t-FMA-structured film and facing electrodes (see the Experimental Section and Figs. S1–S4 for detailed fabrication processes and characterizations). The t-FMA with built-in tilted magnetic moment after magnetization can serve as the flexible permanent magnets. Actuated by the magnetic torque, the t-FMA can be bidirectionally deflected regarding different poles of magnetic field, which causes the variation in electrode separations and hence capacitance signals of the sensor into two non-overlapping regions. Therefore, the sensor can not only perceive the magnitude of magnetic field with different capacitance intensities, but also recognize the orientation of magnetic field with non-overlapping signals (i.e., positive and negative capacitance). Based on this feature, the North pole, South pole, and absence of external magnetic field can be encoded as ‘ + 1’, ‘−1’, and ‘0’ to conveniently form a ternary information coding system (Fig. 1c). Compared with simply turning on/off the external stimuli, the ternary coding system enables the magnetic field as a high-capacity information carrier for efficient communication in HMI system. With the invisibility of magnetic field, a large amount of cryptic information can be therefore encoded in the magnetic fields of a small-scale integrated magnet array (either flexible permanent magnets or electromagnets). Taking advantage of the recognition of both magnitude and orientation of magnetic field, the sensor array can then serve as a decoder to analyze the embedded codes for further information extraction without the high demand on numerous sensor units. From these perspectives, the proposed flexible system can realize the touchless, efficient, and convenient transmission/communication of cryptic information in HMI.

### Optimization on the Actuation of the t-FMA under External Magnetic Field

**Fig. 2:**
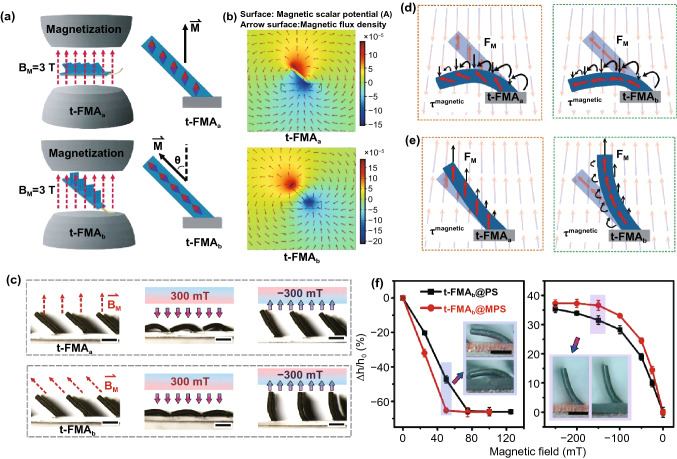
**a **Magnetizing process illustration of t-FMA_a_ and t-FMA_b_. **b** Simulation results of magnetic field distribution of t-FMA_a_ and t-FMA_b_. **c** Actuated deformation of t-FMA_a_ and t-FMA_b_ under the positive and negative magnetic fields with a magnitude of ~ 300 mT. Scale bars: 1 mm. **d-e** Mechanism of the bending actuation of t-FMA_a_ and t-FMA_b_ under positive (**d**) and negative magnetic field (**e**). ***F***_***M***_ and *τ*^*magnetic*^ represent the magnetic force and magnetic torque, respectively. **f** Actuated deformation of t-FMA_b_@PS with PDMS substrate and t-FMA_b_@MPS with NdFeB/PDMS substrate under magnetic fields. Scale bars: 1 mm. h and h_0_ represent the resultant and initial vertical distance from pillar top to the substrate, respectively

As mentioned above, the non-overlapping capacitance signals originally come from the bidirectional deflection of t-FMA under different orientations of magnetic field. The magnetic actuation of t-FMA is thus critical to realize the desired communication system. Herein, we firstly studied the influence of magnetization on the actuation of the t-FMA, as shown in Fig. 2a. The as-prepared titled NdFeB/PDMS micro-pillars (with constant tilted angle of 45° and NdFeB content of 61.5 wt%) upon PDMS substrates were magnetized either in the out-of-plane magnetizing field (i.e., t-FMA_a_) or in the tilted field directed along the micro-pillars (i.e., t-FMA_b_). The magnetizing field, **B**_**M**_, possessed the constant magnitude of ~ 3 T in this work. After magnetization, the micropillars can act as micromagnets with the fixed magnetic moments aligned with the magnetizing field [26, 27]. Such a statement can also be revealed by the simulation results of magnetic field distribution of t-FMA_a_ and t-FMA_b_ (Fig. 2b; detailed simulation can be referred to Section S2 in supporting information). The difference of magnetic moment alignments (i.e., magnetization vector${\varvec{M}}$) led to the different deformations of t-FMA_a_ and t-FMA_b_ when exposed to the out-of-plane magnetic field, as shown in Figs. 2c and S5. For convenient statement, the external magnetic fields with North pole and South pole exerted onto the samples were labelled as positive and negative magnetic field, respectively. The obvious downward bending of both t-FMA_a_ and t-FMA_b_ could be observed when exposed to the positive magnetic field of ~ 300 mT. However, the t-FMA_b_ exhibited the much higher upward deformation than the t-FMA_a_ when exposed to the negative magnetic field of -300 mT. Such results can be attributed to the different interactions between the magnetization (${\varvec{M}}$) of t-FMA_a_ and t-FMA_b_ and the applied magnetic field (${\varvec{B}}$). In general, a high-aspect ratio magnetic material will experience both a magnetic torque ${\varvec{\tau}}={\varvec{M}}\times {\varvec{B}}$ that tends to align it with an applied field and a magnetic force ${\varvec{F}}=\nabla \left({\varvec{M}}\bullet {\varvec{B}}\right)$ that pulls it along an applied field gradient to minimize the Zeeman energy [28–31]. The spatial profile of the magnetic field in Fig. S6 indicates that the field components in x–y plane (i.e., ${{\varvec{B}}}_{\mathrm{xy}}$) applied to t-FMA_a_ and t-FMA_b_ are approximately uniform. However, the magnetic field component in normal direction (i.e., along the central axis of the magnet, ${{\varvec{B}}}_{\mathrm{z}}$) varies with the distance from the magnet surface, which results in the field gradient, $\nabla {{\varvec{B}}}_{\mathrm{z}}$. Therefore, both the magnetic torque and magnetic force may take effect on the actuation of t-FMA in our case. To clarify the dominated actuation, we herein compared the respective energy regarding magnetic torque and magnetic force that rotate t-FMA over a small angle of$\alpha$, that is,${U}_{\tau }/{U}_{F}=B/\nabla BL$, where ${U}_{\tau }\sim MB\alpha$ and ${U}_{F}\sim M\nabla B\alpha L$ are the energy corresponding to the magnetic torque and magnetic force, respectively, and $L$ is the length of the micro-pillars (t-FMA) [31]. It was found that the ${U}_{\tau }$ was at least five times greater than the ${U}_{F}$ across the full scale of magnetic field, indicating that the bending actuation of t-FMA was initially dominated by the magnetic torque in our case (Fig. S7). It is worth noting that the magnetic force also takes effect on the actuation during the bending of t-FMA. When the magnetization of t-FMA becomes more aligned with the applied field, the magnetic force will increase whereas the magnetic torque will decrease (Fig. 2d, e). As the magnetic force possesses the similar impact on the bending actuation, and the initial actuation is dominated by the magnetic torque, we will mainly discuss the magnetic torque for simplification in the following section. For the t-FMA_a_ and t-FMA_b_ exposed to the positive magnetic field, the alignment of their magnetizations was significantly different from the applied field, which induced a large magnetic torque to bend their bodies (Fig. 2d). As a result, both t-FMA_a_ and t-FMA_b_ could be obviously deformed downward. When the negative magnetic field was exerted, however, the t-FMA_a_ originally possessed the consistent alignment between its magnetization and the applied field, and no magnetic torque could be generated at this stage (Fig. 2e). The slight upward deformation of t-FMA_a_ could thus be attributed to the magnetic force which pulled it along the applied field gradient. In comparison, the magnetic torque could still be generated to obviously bend the t-FMA_b_ because of the inconsistent alignment between the magnetization and the applied field. We also especially examined the deformation of non-magnetized t-FMA, as shown in Fig. S8. Due to the absence of fixed magnetization vector (and hence magnetic torque), no obvious bending behavior of the non-magnetized t-FMA can be observed even under the similar magnetic field of $\pm$ 300 mT. With the excellent bidirectional deformation capability, the t-FMA_b_ was selected in the following study.

We also examined the influence of tilted angle (e.g., 0°, 15°, 30°, and 45°) and NdFeB content (15, 50, and 61.5 wt%) on the deformation capability of t-FMA_b_, as shown in Fig. S8. The increased deformation of t-FMA_b_ was observed with the increase in tilted angle and NdFeB content under both the positive and negative magnetic fields (with constant magnitude of 300 mT). Such results can be attributed to the enhanced magnetic torque. The increase in tilted angle gives rise to a greater difference between alignments of the magnetization and the applied field, while the increase in NdFeB content contributes to a higher remanent magnetization [32]. Both increase in the tilted angle and NdFeB content result in the increase in magnetic torque. The influence of NdFeB content on the elastic stress that resisted the bending of t-FMA_b_ might be negligible, because the bending of t-FMA_b_ would result in the restricted elastic compression, while the obviously increased elastic modulus of NdFeB/PDSM with NdFeB content was observed under large compressions (Fig. S9). We herein employed the tilted angle of 45° and NdFeB content of 61.5 wt% in this work because the samples with greater tilted angle and NdFeB content are difficult to fabricate. Note that the influence of diameter, length and density of micro-pillars was not especially studied in this work. In principle, a higher aspect ratio (i.e., lower diameter and higher length) contributes to a greater deformation capability of micro-pillars. We herein selected the minimum diameter of 300 μm and maximum length of 1.7 mm (corresponding to a vertical height of 1.4 mm for t-FMA_b_ with a tilted angle of 45°) because the smaller diameter and higher length are difficult to fabricate based on the current approach. It is also worthwhile to note that the t-FMA is required to assemble with electrodes to form a stable sensor, where the binding effect may produce additional elastic stress to restrict the deformation of the assembled t-FMA. A relatively higher density of micro-pillars may conduce to a globally greater driving force to ensure the effective bending actuation of the assembled t-FMA. However, the micro-pillars with excessive density may still suffer from the restricted deformation capability because the adjacent micro-pillars will contact mutually during the deformation. The maximum density (i.e., minimum edge distance of adjacent pillars) is therefore determined by the length of micro-pillars. As mentioned above, the micro-pillars of the optimized t-FMA_b_ with the tilted angle of 45° possess the length of ~ 1.7 mm, and thus the corresponding maximum density (i.e., minimum edge distance of adjacent pillars of ~ 1.7 mm) was selected in this work. As the selected dimension parameters are already optimal in our case, we herein introduced the NdFeB/PDMS substrate (thickness of ~ 500 μm) with equal NdFeB content (61.5 wt%) instead of the PDMS substrate to further enhance the deformation capability of t-FMA_b_. For convenient statement, the samples with NdFeB/PDMS substrate and PDMS substrate were labelled as t-FMA_b_@MPS and t-FMA_b_@PS, respectively. After similar magnetization process, the t-FMA_b_ with NdFeB/PDMS substrate (i.e., t-FMA_b_@MPS) exhibited the higher deformation than the one with PDMS substrate (i.e., t-FMA_b_@PS) when exposed to the external magnetic field (Fig. 2f). Such results can also be attributed to the increased remanent magnetization of t-FMA_b_@MPS and hence the enhanced magnetic torque, as indicated by the hysteresis loops in Fig. S10. Compared with t-FMA_b_@PS, the t-FMA_b_@MPS possessed the higher amount of NdFeB particles in the similar volume, corresponding to a higher NdFeB content and hence a greater remanent magnetization [32]. As a result, the magnetic torque could be enhanced, which contributed to the higher deformation capability of t-FMA_b_@MPS. With the excellent deformation capability, the t-FMA_b_@MPS was then selected as the dielectric layer for the capacitive sensor of magnetic field in this work.

### Sensing Performance of the Capacitive Sensor of Magnetic Field

**Fig. 3:**
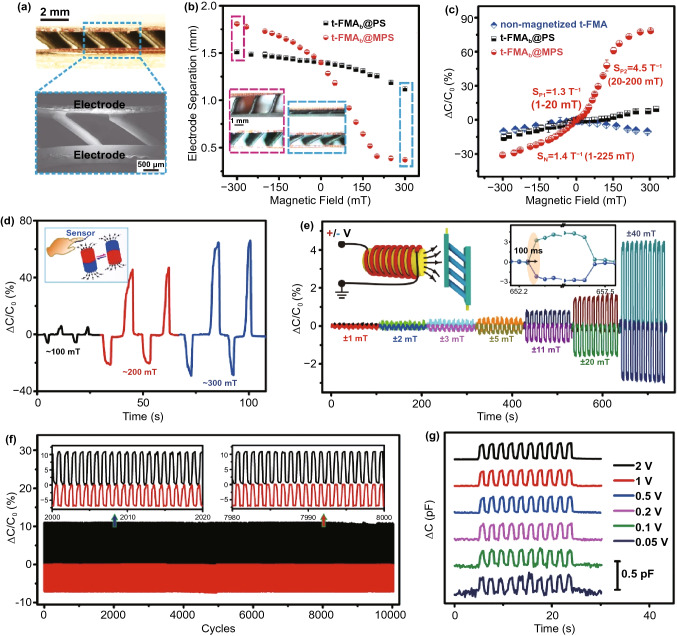
**a **Side-view of optical and SEM images of the assembled sensor based on t-FMA_b_@MPS. **b** Variations of the electrode separation of the assembled device based on t-FMA_b_@PS and t-FMA_b_@MPS under magnetic fields. **c** Relative capacitance variation of the sensor based on t-FMA_b_@PS, t-FMA_b_@MPS and non-magnetized t-FMA_b_@MPS under magnetic fields. **d** Real-time response of the optimized sensor to different magnetic fields. **e** Stability of the sensor under different magnitudes of electromagnetic field. **f** Long-term stability of the sensor under the periodic electromagnetic field of ~ 65 mT for over 10,000 cycles. **g** Electric response of the sensor to electromagnetic field of ~ 65 mT under different operating voltages

To form the stable sensor devices, the optimized t-FMA_b_@MPS dielectric layer was assembled with the facing copper fiber electrodes by introducing a PDMS layer that jointed the electrode and the apex of micro-pillars, as shown in Fig. 3a (detailed assembling process can be referred to the Experimental Section). The binding effect of the PDMS layer that connects the micro-pillar apex and the electrode may produce the additional elastic stress that restricts the bending behavior of t-FMA when exposed to the external magnetic field. Therefore, the magnetic field-induced deformation of the assembled t-FMA_b_@MPS was dramatically decreased when compared with that of the unassembled one, as indicated in Figs. 2f and 3b. However, the t-FMA_b_@MPS still exhibited the much higher deformation than the t-FMA_b_@PS in accordance with the results in Fig. 2f, revealing that the magnetic torque can still effectively result in the micropillar bending for the assembled device. Figure 3c presents the relative capacitance variation of sensors based on t-FMA_b_@PS and t-FMA_b_@MPS exposed to the positive and negative magnetic fields. Owing to the higher deformation capability of t-FMA_b_@MPS, the sensor based on t-FMA_b_@MPS exhibited the better sensing performance, with the high sensitivity of 1.3 T^−1^ (1–20 mT) and 4.5 T^−1^ (20–200 mT) under positive magnetic field, and 1.4 T^−1^ (1–225 mT) under negative magnetic field (Figs. 3c and S11). Here, the sensitivity is given by $S=\frac{\partial \left(\Delta C/{C}_{0}\right)}{\partial B}$, where ${C}_{0}$ is the initial capacitance before applying magnetic field, $C$ the resultant capacitance under magnetic field and $B$ the magnetic flux density of the applied field [33–36]. According to the definition of capacitance ($C={\varepsilon }_{0}{\varepsilon }_{\mathrm{r}}A/d$, where ${\varepsilon }_{0}$ is the permittivity of vacuum, ${\varepsilon }_{\mathrm{r}}$ the relative dielectric constant, $d$ the distance between the facing electrodes and $A$ the facing area between electrodes (constant in this work)), both the variation in ${\varepsilon }_{\mathrm{r}}$ and $d$ can affect the capacitance variation. In our case, the decrease (or increase) of $d$ is also accompanied with the increase (or decrease) of ${\varepsilon }_{\mathrm{r}}$, which consistently contributes to the increase (or decrease) of capacitance, as discussed in Fig. S12. Therefore, the capacitance variation can be mainly attributed to the deformation of t-FMA_b_ from the bending actuation when the sensor is exposed to external magnetic field. With the competitive deformation capability of t-FMA_b_@MPS, the sensor based on t-FMA_b_@MPS can exhibit the better sensing performance, and therefore is proposed in this work. We also especially examined the relative capacitance variation of the sensor based on the non-magnetized t-FMA_b_@MPS for comparison (Fig. 3c). Without the magnetization and hence the magnetic torque, the bending actuation could not occur within the non-magnetized t-FMA_b_@MPS, which resulted in the failed response to external magnetic fields. However, the magnet approaching affected the spital charge distribution (namely, the proximity sensing) [37–39], which caused the similar decreased capacitance under both positive and negative magnetic field. It is worth noting that the capacitance variation caused by proximity sensing is negligible within the magnetize devices, which also reveals that our proposed sensor can be reliably used for detecting magnetic fields even with the presence of object approaching.

Figure S13 presents the sensing performance of our sensor across different samples. The repeated curves of capacitance variation reflect the excellent reproducibility of our proposed sensor and methodology. The real-time detection of external magnetic fields was confirmed by moving the sensor close to or away from different poles of the magnet, as shown in Fig. 3d. The non-overlapping capacitance signals could be observed when the positive and negative magnetic field was separately exposed to the sensor, and the signal intensity changed in accordance with the magnitude of magnetic field. Such results indicate that our proposed sensor can recognize both the orientation and magnitude of external magnetic fields in real-time. We also noticed that the generation of non-overlapping capacitance signals is also available when the magnetic field is applied parallel to the proposed sensor (Fig. S14). Attributed to the tilted magnetization vector of the t-FMA_b_@MPS, the different alignments between the magnetization and the applied magnetic field still exist. The induced magnetic torque can thus bidirectionally bend the t-FMA_b_@MPS under positive and negative magnetic field, resulting in the similar non-overlapping capacitance signals of the sensor. Such results also reveal that our proposed sensor does not strictly rely on the out-of-plane magnetic field (i.e., perpendicular to the sensor) to generate non-overlapping signals for information communication, which can promise the convenience in real applications. We also evaluated the sensing stability of the sensor under different magnitudes of magnetic field induced by the electromagnet, as shown in Fig. 3e (the regulation of orientation and magnitude of the magnetic field can be referred to Fig. S15). The non-overlapping capacitance signals could be well repeated under each field magnitude, indicating the excellent stability of our sensor for magnetic field sensing. Moreover, the sensor exhibited the fast response/relaxation time of ~ 100 ms, as indicated by the inset of Fig. 3e. Such a result reveals that our proposed sensor can rapidly convert the magnetic field variation to capacitance signals, which can be potentially used for the rapid information conversion/transmission if employing the magnetic field as an information carrier. Video S1 further presents the dynamic deformation of the sensor under different orientations of magnetic field (with constant magnitude of 40 mT). The rapid and stable deformation of t-FMA_b_@MPS could be observed under the bending actuation of magnetic torque, which indicates that the stability and fast response/relaxation of the sensor originally come from the excellent elasticity of t-FMA_b_@MPS. Taking advantage of the high sensitivity, the sensor also exhibited the excellent detection limit down to 1 mT, revealing that our proposed sensor is capable of recognizing the tiny variation in magnitude of magnetic field (Fig. 3e). To clarify the possible interference of electric field generated by the electromagnet, we also examined the real-time response of the sensor based on the non-magnetized t-FMA_b_@MPS (Fig. S16). The unchanged signal reflects that the capacitance variations of our proposed sensor completely come from the response to magnetic field. The long-term stability of the sensor was also confirmed by periodically applying the positive and negative magnetic field (with the constant magnitude of 65 mT and the period of 1 s) for over 10,000 cycles, as shown in Fig. 3f. The well-repeated capacitance signals indicate the excellent stability and accuracy of our proposed sensor towards long-term applications.

We also evaluated the possible influence of the global bending on the sensing performance of the sensor (Fig. S17). The unchanged capacitance signals under different bending degrees reveal that the electric response to magnetic field of our proposed sensor is not affected by the slight bending of sensors as wearable device. Figures 3g and S18 present the capacitance variation of sensor exposed to the positive and negative magnetic field (magnitude of 65 mT) with different operating voltages. The capacitance signals could still be repeated even under a low operating AC voltage of 50 mV, which reflects the extremely low energy consumption of our proposed sensor. The influence of operating frequency of the AC voltage on the performance of the sensor was also evaluated, where the stable electric response to magnetic field could be observed with a frequency over 1 kHz (Fig. S19). With the advantages of high sensitivity, magnetic poles distinguishability, facile fabrication, low-energy consumption, etc., our proposed sensor is therefore competitive to many reported magneto-sensitive smart skins including anisotropic magnetoresistance (AMR), giant magnetoresistance (GMR), magneto-impedance (GMI) and Hall sensor [21, 40–45]. To our knowledge, AMR, GMR, and GMI sensors mostly fail to recognize the magnetic poles because they produce the monotonous (positive) electric signal under each pole of the magnetic field [46, 47]. The Hall sensors can perceive both the magnitude and orientation of magnetic field with non-overlapping signals, while the high operating DC current and precise fabrication are normally demanded. In comparison, our proposed capacitive sensor of magnetic field possesses the natural advantages of low-energy consumption and ease of fabrication as mentioned above. The detailed comparison results regarding the sensing performance, fabrication method, operating condition, etc. are presented in Table S1. Besides the magnetic field sensing, our proposed sensor also possesses the additional function of tactile sensing in contact mode when loading pressures onto the sensor (Fig. S20). Based on the optimized t-FMA_b_@MPS, the sensor exhibited the high sensitivity of 0.38 kPa^−1^ (0–2 kPa) and the broad working range over 200 kPa. The excellent elasticity of t-FMA_b_@MPS also enabled the repeated variation curves of capacitance signal without obvious hysteresis during the dynamic pressure loading/unloading test. Moreover, the sensor also exhibited the excellent stability under different pressure intensities and the excellent durability towards long-term and high-pressure applications (Figs. S21 and S22). The successful monitoring of daily mechanical stimuli, e.g., ultralight substances including rice and paper, airflows, finger presses, and wearable applications (such as pulse sensing and voice recognition), also reveals the potential of our device for applications in soft robotic, health care monitoring and so on (Figs. S23 and S24). It is worth noting that such an additional function of tactile sensing does not affect the magnetic field sensing of our sensor, because they take effect in contact mode and touchless mode, respectively. Once the pressure is loaded, the tactile sensing will dominate the capacitance signal of the sensor as the pressure loading gives rise to the much greater capacitance variation than the magnetic field (Figs. 3c and S20). With the functions of both magnetic field sensing and tactile sensing, it is expected that our proposed sensor can serve as a multifunctional wearable device to adapt the diverse applications.

### Fast and Efficient Morse Code and Braille Communication System

**Fig. 4:**
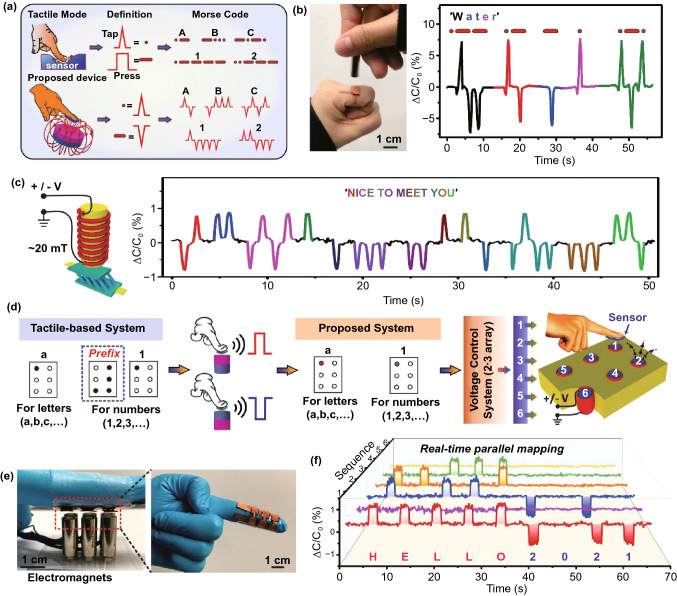
**a **Schematic of touchless Morse code communication by simply switching the orientation of magnetic field based on our proposed sensor. **b** Real-time monitoring of the Morse code transmission of ‘water’. **c** Programmable Morse code transmission of ‘Nice to meet you’. **d** Schematic of the touchless, efficient, convenient, and programmable Braille interaction system based on our proposed sensor. **e** Photograph of the proposed Braille interaction system. **f** Real-time mapping of capacitance signals regarding the electromagnetic fields to decode the Braille information of ‘HELLO 2021’

With the ability of recognizing the orientation of magnetic field (i.e., North/South poles), our proposed sensor can serve as a wearable transmitter for the touchless Morse code communication by simply switching the orientation of magnetic field instead of the fast tapping and long-time pressing in contact mode (Fig. 4a). Specifically, the North or South pole of external magnetic field can be utilized as the Morse codes of ‘dot’ or ‘dash’ to encode the desired information. As the sensor can generate the positive and negative capacitance signal regarding the North and South poles, the encoded information can be transmitted and decoded by the combinatorial non-overlapping capacitance signals. Figure 4b presents the real-time Morse code transmission of ‘water’ by regularly switching the North/South pole of a permanent magnet (with magnitude of ~ 200 mT) exposed to the sensor (the dynamic monitoring can be referred to Video S2). The positive and negative capacitance signal corresponding to the ‘dot’ and ‘dash’ can be clearly distinguished to decode the information, which indicates the potential of our proposed sensor for touchless and convenient Morse code communication. Similar transmission of ‘water’ using the fast tapping and long-term pressing mode was also examined for comparison (Fig. S25), which convinces the superiority of time-saving and efficiency based on our proposed Morse code system. Such a Morse code interaction system can be used as a tool to facilitate the daily life of special groups. For example, it can be applied to help patients communicate with doctors more effectively about their situations when they have difficulty in speaking and writing. Our proposed sensor is also applicable for the programmable Morse code transmission, where the input magnetic fields can be programmed using the electromagnet, as shown in Fig. 4c. Here, the electromagnet was placed with a distance of ~ 2 mm above the sensor and the generated magnetic field exhibited the constant magnitude of ~ 20 mT. By pre-defining the waveform of the voltage supply, we can get the real-time capacitance signals and recognize the desired message of ‘nice to meet you’ after the decoding process. The dynamic monitoring of information transmission and the designed waveform of output voltage can be referred to Video S3 and Fig. S26, respectively. We also mimicked the conventional tactile recognition system via simply tuning the duration of voltage applied on the electromagnet without alternating the magnetic poles for comparison (Fig. S27). Similar to the tactile-based system, such an approach required a longer duration to deliver the information carried by Morse code when compared with our proposed system. Figure S28 further present the overall map of the representative alphabet and numbers transmitted by our proposed Morse code interaction system. The results herein sufficiently indicate the potential of our proposed sensor for the touchless, efficient, and convenient Morse code transmission.

Braille, comprised of rectangular cells with special dot patterns, is an important communication tool for visually impaired people [47, 48]. For tactile-based Braille system, the additional cell called ‘numbers prefix’ is always required to differentiate the letters and numbers because they have similar dot patterns (Fig. 4d). Such an additional cell, however, can be avoided if employing our propose sensor for touchless Braille interaction. As the proposed sensor can respond to different orientations of magnetic field with non-overlapping capacitance signals, it is feasible to optimize the overall system using the contrary signals for letter or number definition. Without the dependence on ‘numbers prefix’, the reading time of Braille can be reduced with improved recognition/communication efficiency, and the construction burden can also be relieved especially for wearable applications. Without direct touching, the interference of the pseudo signals resulted from invalid contact in tactile-based Braille system can also be avoided. Moreover, it is possible to realize the programmability of information monitoring in an efficient and convenient way using the magnetic field as information inputs based on the programmable electromagnet. The proposed touchless and programmable Braille interaction system is presented in Fig. 4e, where a 2 × 3 sensor array (with the size of 0.5 × 0.5 cm^2^ for each sensor pixel) was attached on the finger of a volunteer above the electromagnet array (with a distance of ~ 2 mm) via adhesive tapes to connect the edge of sensor array with the finger. By supplying positive or negative voltages (with constant magnitude of 12 V) for each electromagnet, the magnetic field arrangements can be easily programmed to encode the desired information. Figure 4f presents the real-time capacitance signals received by the sensor array, where the Braille information 'HELLO 2021' could be obtained after decoding a series of signals (the programmed voltage groups for the magnetic field outputs can be referred to Fig. S29). These results indicate that our device can realize the real-time detection of the programmable braille information based on a single 2 × 3 array, which can greatly improve the efficiency of Braille transmission/readout for the convenient and fast communication. Thanks to the distinguishability to magnetic poles, the proposed system does not rely on the ‘prefix’ to differentiate numbers and letters, revealing the possibility of construction burden reduction as well as the efficiency optimization. Note that the high sensitivity of the sensor is another important prerequisite to realize the localized perception of a weak magnetic field, which thus relieves the potential hazard regarding the wearable and daily applications.

### Ternary System for Cryptic Information Communication and Contactless HMI Application

**Fig. 5:**
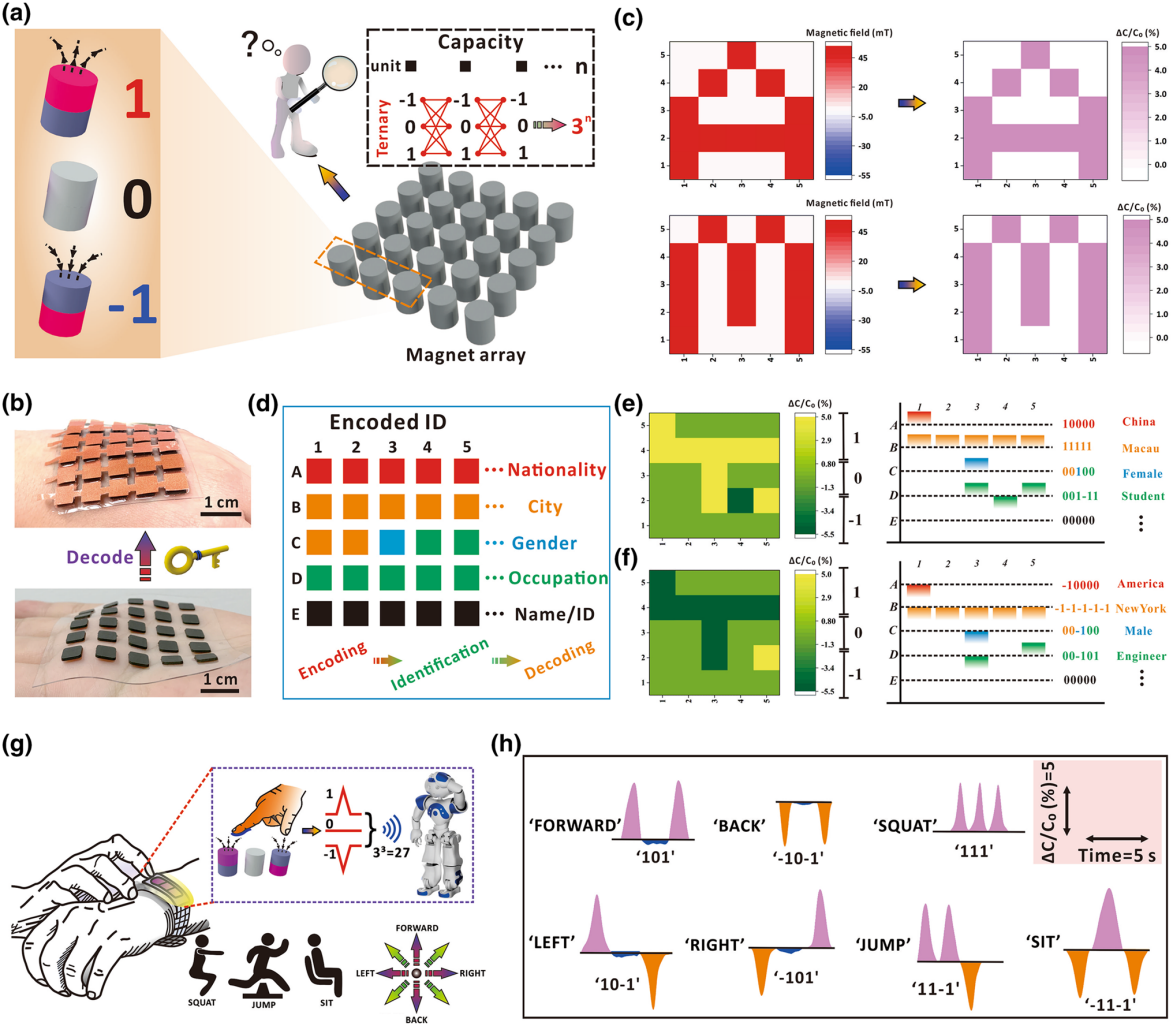
**a **Schematic diagram of the ternary information coding system to encode cryptic information. **b** Demonstrated flexible magnet array and sensor array. **c** Mapping of magnetic fields customized in the flexible magnet array and the capacitance signals obtained by the sensor array with cryptic letters of ‘A’ and ‘M’. **d** Proposed cryptic ID recognition system. **e–f** Obtained capacitance signal mapping and the decoded ID information. To avoid the experimental errors, the relative capacitance variation below -2%, in the range of -2% to 1.5%, and over 1.5% was decoded as ‘-1’, ‘0’, and ‘1’, respectively. **g** Proposed high-capacity transmitter for outputting multi-bit coded control instructions based on our sensor. **h** 3-bit coded instructions transmitted by the sensor

Taking advantage of the recognition of both orientation and magnitude of magnetic field, the North pole, South pole, and absence of external magnetic field can be further encoded as ‘ + 1’, ‘-1’, and ‘0’ to conveniently form a ternary information coding system (Fig. 5a). Compared with simply turning on/off the external stimuli (i.e., binary coding system of ‘1’ and ‘0’), the ternary coding system enables the magnetic field as a high-capacity information carrier for efficient communication in HMI system. With the invisibility of magnetic field, a large amount of cryptic information can therefore be encoded in the magnetic fields using a small-scale integrated flexible magnet array. Then the sensor array can serve as a decoder to analyze the embedded codes for further information extraction without the high demand on numerous sensor units. Figure 5b presents the demonstrated flexible magnet array and sensor array for cryptic information transmission, where both the magnet array and sensor array consist of 5 × 5 units (with the unit area of 0.5 × 0.5 cm^2^; detailed fabrication of the flexible magnet array can be referred to experimental section). The sensor array was obtained by assembling the proposed t-FMA structures between the crossed electrodes, where the crossed electrodes were obtained by arranging 5 upper electrodes in rows and 5 bottom electrodes in columns to form 25 facing regions (Fig. S30). The assembly between the t-FMA structures and electrodes was realized by introducing a PDMS layer that jointed the electrode and the apex of micro-pillars as described in Fig. 3a and the experimental section. The recognition of hidden patterns, e.g., cryptic letters of ‘A’ and ‘M’ was firstly demonstrated, as shown in Fig. 5c. The letter information was encoded by customizing the magnetic field of specific magnet pixels, and was decoded from the capacitance signals received by the sensor array. The well-identified relative capacitance variations consistent with magnetic field distribution reveal that our proposed device possesses great potentials for cryptic information transmission. Figure S31 also presents the decoded letters of ‘A’ and ‘M’ customized with the reverse orientation of magnetic fields, which indicates the capability of our device for the diverse transmission of cryptic information. Based on the ternary information coding of magnetic field, a large amount of cryptic identity information, e.g., nationality, gender, name, and occupation, can also be encoded in the magnetic fields of a small magnet array with 5 × 5 pixels, as indicated in Fig. 5d. After scanning the magnet array by the sensor array, a series of capacitance signals could be obtained in accordance with the customized magnetic fields, as shown in the left column of Fig. 5e, f (the customized fields can be referred to Fig. S32). By decoding the received capacitance signals, the pre-defined identity information of ‘a female student from Macau, China’ and ‘a male engineer from New York, USA’ could be recognized, as shown in the right column of Fig. 5e, f. Such results reveal the potential of our proposed device for identifying the encoded identity information. Note that with the invisibility of magnetic field, the encoded information can also be transmitted and identified with security. The facile fabrication and low-energy consumption of our sensor devices are also the advantages that conducts to the overall simplification of the information transmission system in real applications.

The distinguishable recognition of both magnetic field orientation and magnitude also enables our sensor as a high-capacity transmitter to output multi-bit coded control instructions in a touchless way with the ternary coding magnetic fields as stimulus inputs (Fig. 5g). Based on the ternary coding input, there are 27 3-bit coded instructions in total can be transmitted via a single sensor. Typically, these instructions can sufficiently cover and control a variety of motions of the targets (e.g., robots), such as the movement, jumping, and squatting, etc. As a proof-of-concept, we herein demonstrated several encoded groups of ‘101’, ‘−10–1’, ‘111’, ‘10–1’, ‘−101’, ‘11–1’, ‘−11–1’ regarding different instructions, as shown in Fig. 5h. The instruction codes of ‘1’, ‘-1’ and ‘0’ were controlled by exposing the North pole, South pole and absence of magnetic field to the sensor, respectively, and were decoded/transmitted from the corresponding signals of positive, negative and unchanged capacitance perceived by the sensor. The diverse collocations of ternary signals for different instructions indicate the potential of our proposed device as a convenient and effective platform for human–machine interaction system, flexible control device, etc. with multi-functions. Note that these instructions can be transmitted in a touchless way, which may avoid the unprecise instruction transmission caused by the mistouch in contact mode. Moreover, taking advantages of the excellent sensing performance, our proposed sensor (or sensor array) can also be used for detecting the presence of additional magnetic pulse to ensure a secure surrounding prior to the application of information transmission/communication, which can avoid the potential influence of additional magnetic fields.

## Conclusions

In summary, we reported a capacitive sensor of magnetic field based on the t-FMA dielectric for touchless, efficient, convenient, and programmable transmission/communication of cryptic information in HMI. The t-FMA can bidirectionally deform regarding different orientations of magnetic field (i.e., North/South poles) actuated by the magnetic torque, thus enabling the sensor to recognize both the magnitude and orientation of magnetic field with non-overlapping capacitance signals. With the overall optimizations, the sensor exhibits high sensitivity of over 1.3 T^−1^ with detection limit down to 1 mT, response and relaxion time of ~ 100 ms, and excellent durability. Thanks to the swift and sensitive response to magnetic field, the sensor has been demonstrated as efficient and convenient information conversion platform for high-speed, programmable, and touchless interaction system, such as Morse code and Braille interaction. The capability of recognizing both the orientation and magnitude of magnetic field further enables the sensor to decode a large amount of cryptic information, e.g., cryptic letters and ID information encoded in the magnetic fields of a small-scale integrated flexible magnet array. By customizing the magnetic field input, the sensor can also serve as a high-capacity transmitter for multi-instruction outputting for applications in intelligent control system. We expect that the communication interfaces based on our proposed sensor can bring benefits for human-friendly robotics, virtual reality device, and human–machine interaction device, etc. in the future.

## Supplementary Information

Below is the link to the electronic supplementary material.

Supplementary file1 (PDF 2496 kb)

Supplementary file2 (MP4 18307 kb)

Supplementary file3 (MP4 10122 kb)

Supplementary file4 (MP4 1565 kb)
