# Supplementary material for: Magnetized Micropillar-Enabled Wearable Sensors for Touchless and Intelligent Information Communication
Source: Nanomicro Lett. 2021 Sep 14;13:197. doi: 10.1007/s40820-021-00720-5 (PMC8440750; doi:10.1007/s40820-021-00720-5)
Supplement: Supplementary file 1 — Supplementary file1 (PDF 2496 kb) [file 40820_2021_720_MOESM1_ESM.pdf]

Supporting Information for

Magnetized Micropillar Enabled Wearable Sensors for Touchless and Intelligent Information Communication

Qian Zhou[1, #], Bing Ji[1, #], Fengming Hu[2], Jianyi Luo[2], and Bingpu Zhou[1, *]

[1]Joint Key Laboratory of the Ministry of Education, Institute of Applied Physics and Materials Engineering, University of Macau, Avenida da Universidade, Taipa, Macau 999078, P. R. China

[2]Research Center of Flexible Sensing Materials and Devices, School of Applied Physics and Materials, Wuyi University, Jiangmen 529020, P. R. China

# Qian Zhou and Bing Ji contributed equally to this study and share the first authorship

*Corresponding author. E-mail: bpzhou@um.edu.mo (Bingpu Zhou)

Supplementary Tables and Figures

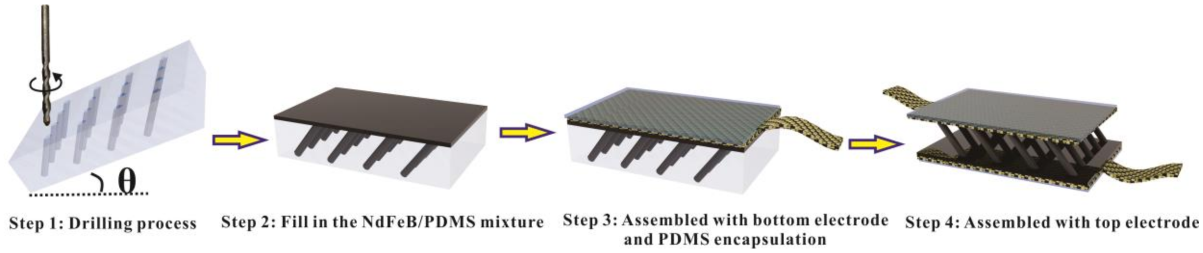

Fig. S1 Schematic of the fabrication process of the capacitive sensor of magnetic field. The template of tilted micropillar array with negative structures was prepared by micro-engraving technique. A polycarbonate (PC) plate, placed on a worktable with adjustable tilted angles, was drilled with hole patterns by a vertical micro drill bit controlled by the micro-engraving machine. The pattern parameters of the micro-holes (e.g., diameter, depth and density) can be flexibly adjusted by changing the size of the drilling bit and the drilling parameters (e.g., drilling depth and spacing).

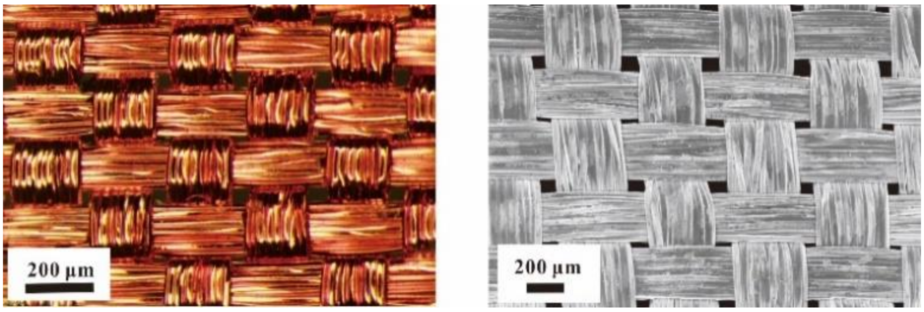

Fig. S2 Optical and SEM images of copper-coated fiber electrode

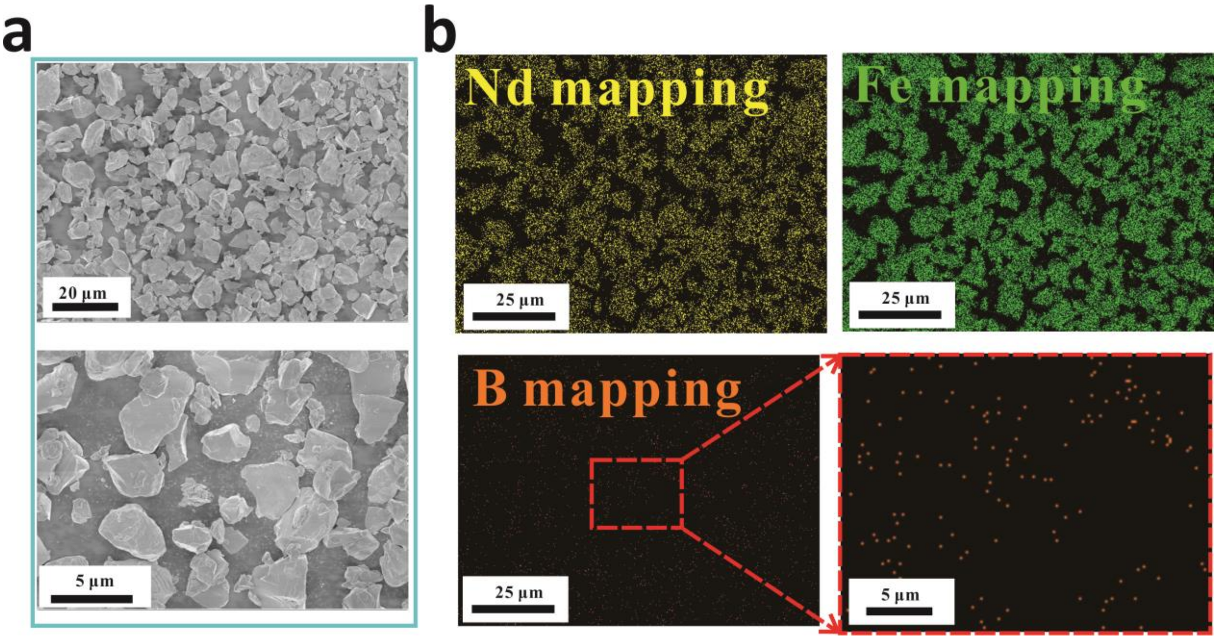

Fig. S3 a) SEM images and **b**) EDX mapping of NdFeB particles. It can be observed that the non-magnetized particles possess an average size of ~5 μm

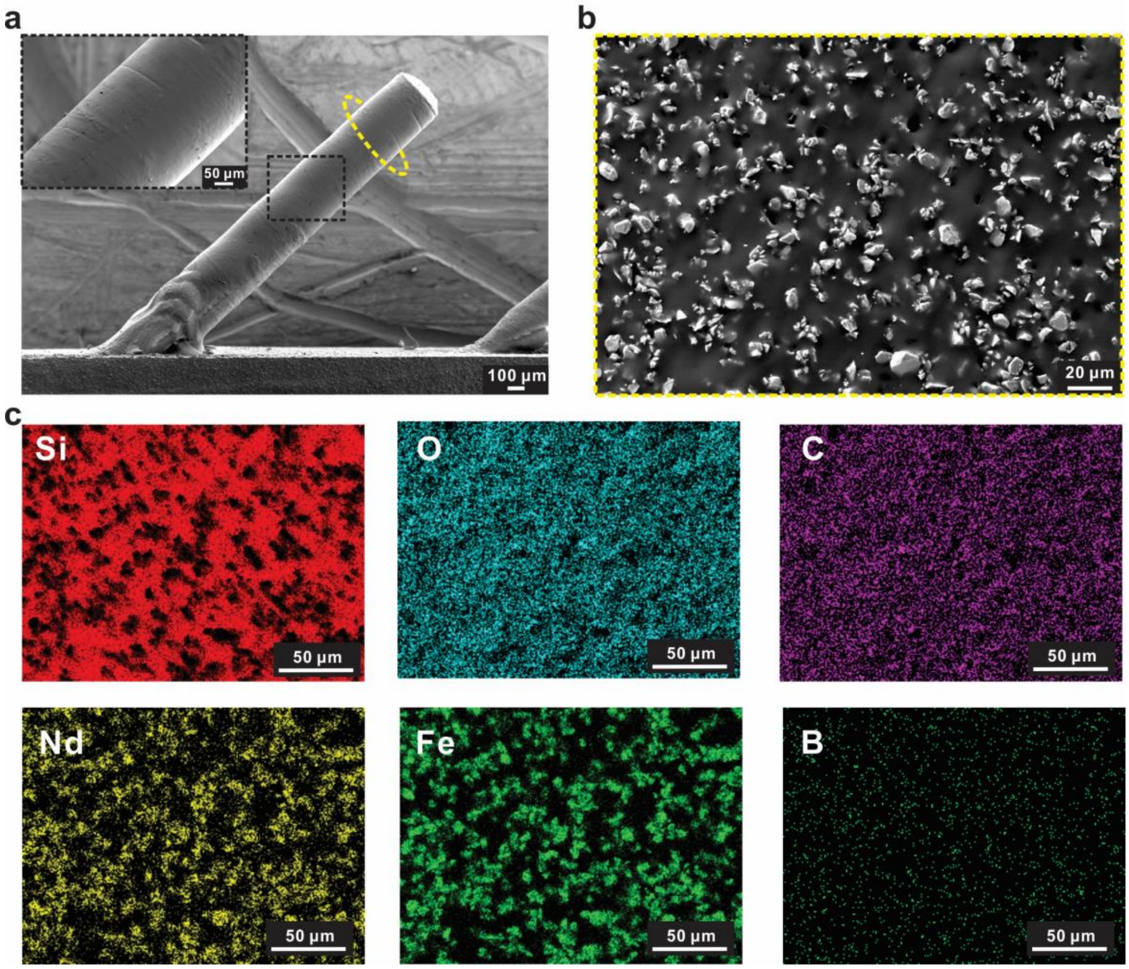

Fig. S4 a) SEM images of magnetic pillar with diameter of 300 μm, tilted angle of 45°, and NdFeB content of 61.5 wt%. **b**) SEM image and **c**) EDX mapping of NdFeB/PDMS obtained from the cross section of the pillar shown in the yellow dotted line in Fig. S4a. The results indicate that the NdFeB particles can be uniformly distributed in PDMS

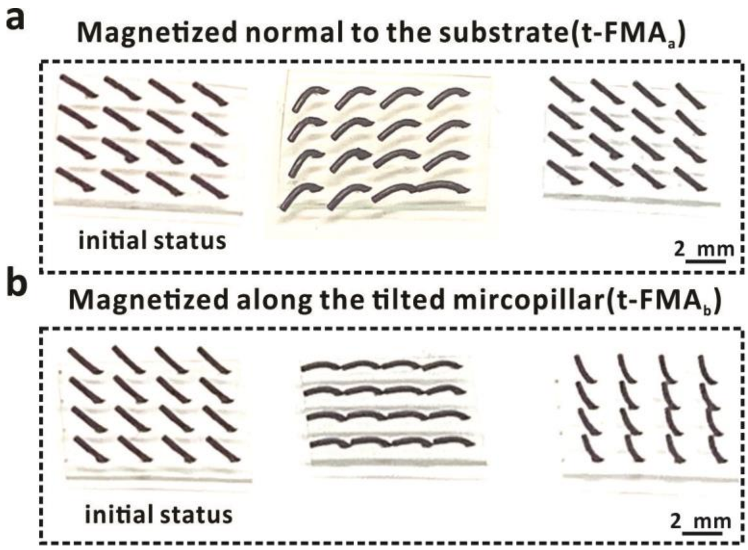

Fig. S5 Dynamic deformation of **a**) t-FMA$_a$ and **b**) t-FMA$_b$ under the external magnetic field of 300 and -300 mT

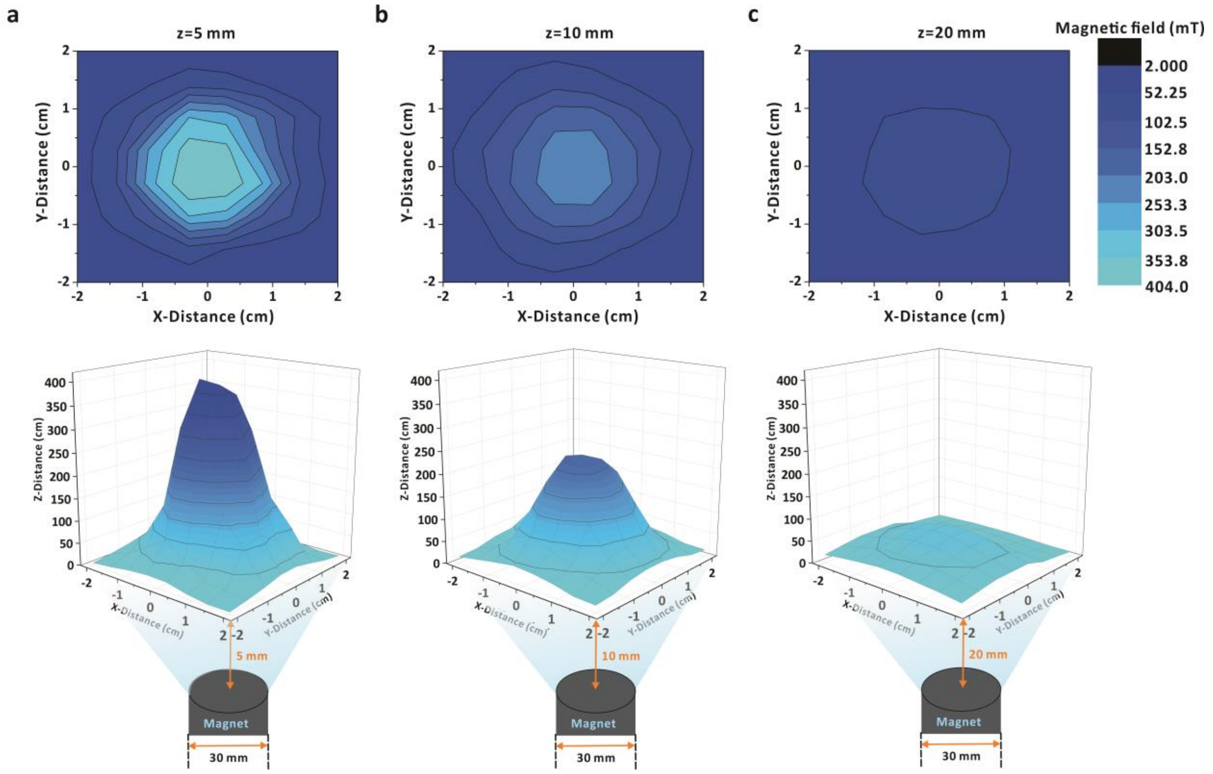

Fig. S6 Magnetic field distribution of the xy-plane at **a**) z = 5, **b**) 10, and **c**) 20 mm above the NdFeB magnet with diameter of 30 mm. As the samples were exposed to central area of 1cm^2 of the magnetic field, the magnetic field components in x-y plane could be regarded as uniform field. However, magnetic field component in normal direction (i.e., along the central axis of the magnet) varied with the distance from the magnet surface, which resulted in the field gradient.

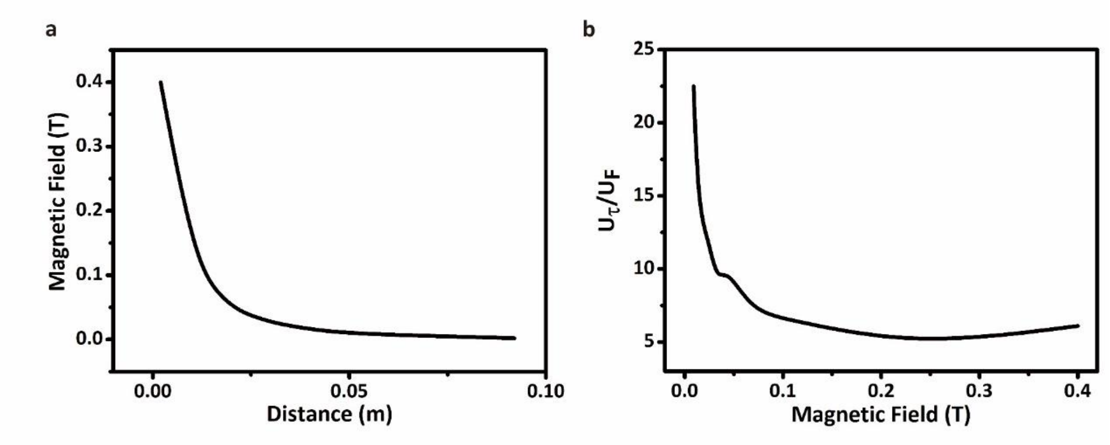

Fig. S7 a) Variation in magnetic flux density of the applied magnetic field with the distance from the magnet surface. **b**) Calculated energy ratio regarding the magnetic torque and magnetic force that rotates t-FMA over a small angle. $U_\tau/U_F = B/\nabla BL$, where $U_\tau \sim MB\alpha$ and $U_F \sim M\nabla B\alpha L$ are the energy corresponding to the magnetic torque and magnetic force, respectively, and L is the length of the micro-pillars of t-FMA (2 mm). The magnetic field gradient was calcualted according to Fig. S6a

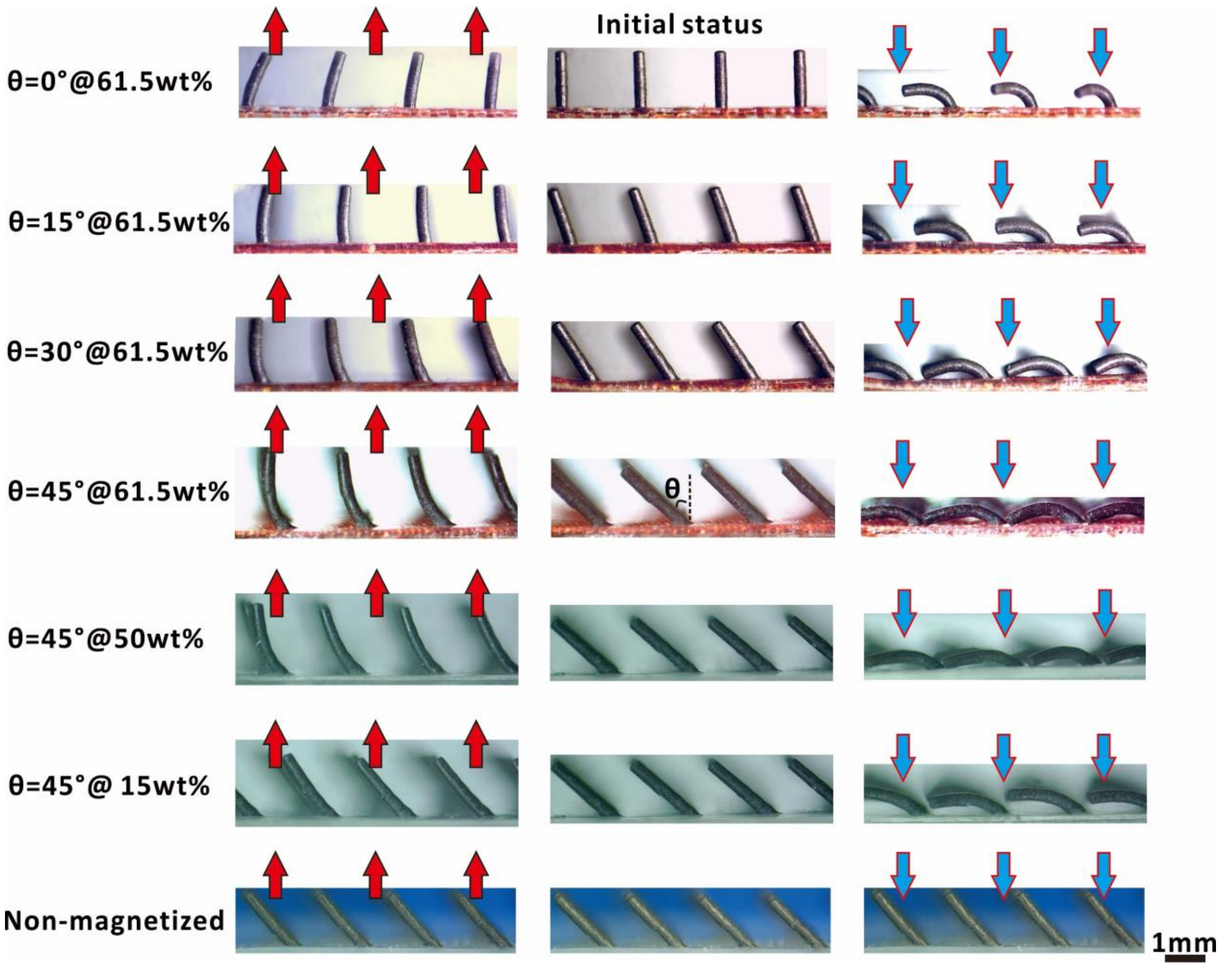

Fig. S8 Dynamic deformation of the t-FMA$_b$ with different tilted angles (0°, 15°, 30° and 45°) and NdFeB content (15wt%, 50wt% and 61.5wt%) as well as non-magnetized t-FMA (tilted angle of 45° and NdFeB content of 61.5wt%) under the magnetic fields of 300 and -300 mT. The tilted angle θ is defined as the angle between pillar and the normal direction.

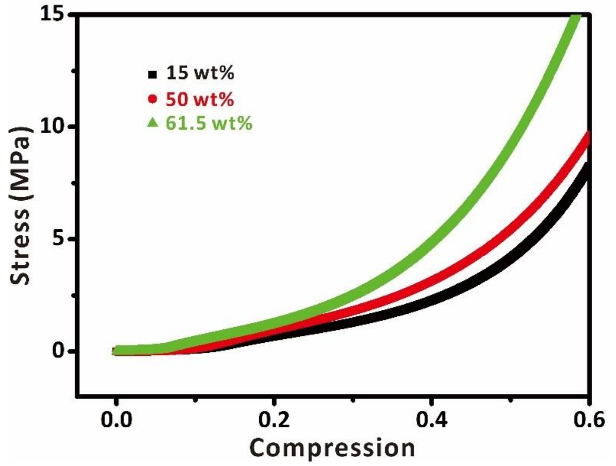

Fig. S9 Stress-compression curve of NdFeB/PDMS with different NdFeB contents (15, 50 and 61.5 wt%)

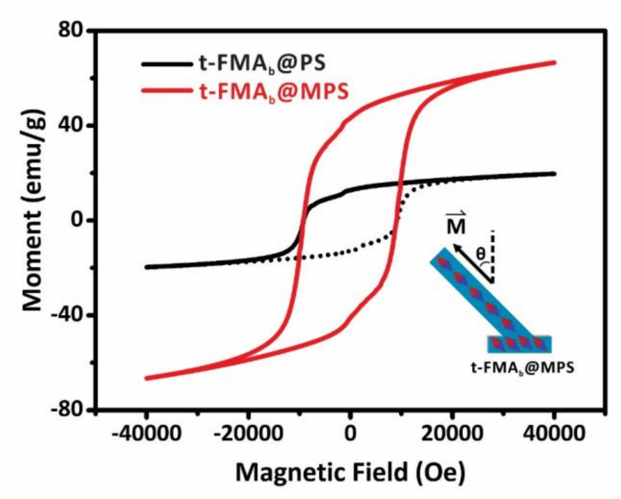

Fig. S10 Hysteresis loops of t-FMA$_b$@PS and t-FMA$_b$@MPS measured at room temperature. The dashed line is symmetrically generated from the solid part of the hysteresis loop for t-FMA$_b$@PS

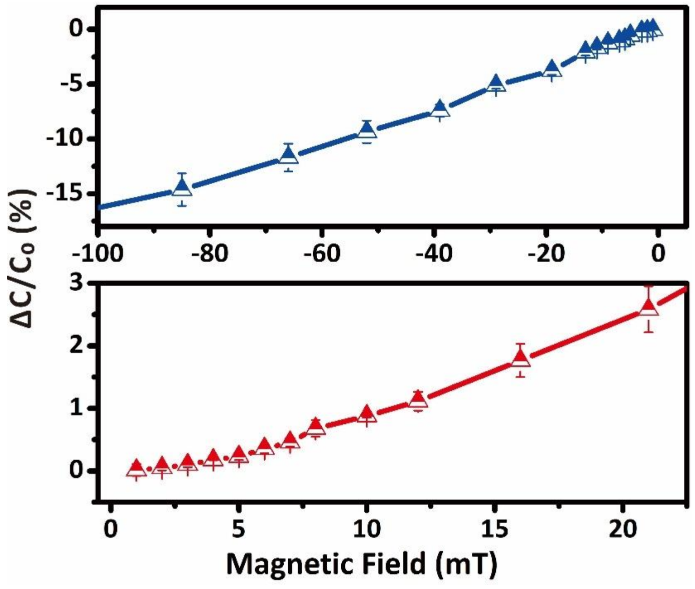

Fig. S11 Enlarged view of the sensor performance curve in the range from 1 to 20 mT (red line) and -100 to -1 mT (blue line) of the applied magnetic field

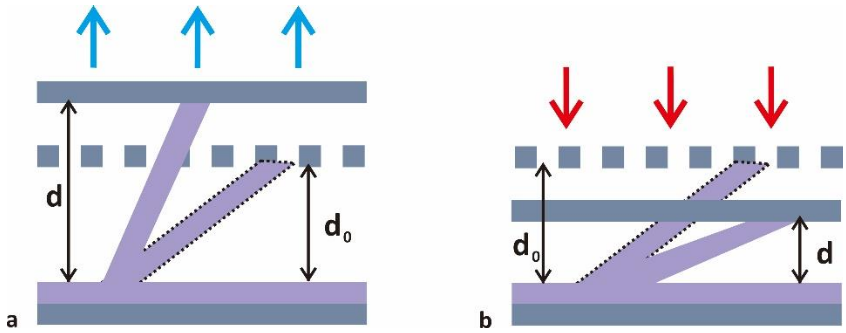

Fig. S12 The deformation schematic of the t-FMA$_b$@MPS before and after applying a certain magnetic field that induces (**a**) upward bending and (**b**) downward bending of the t-FMA$_b$@MPS

The sensitivity of the sensor to magnetic field can be defined by:

$$S = \frac{\partial(\Delta C/C_0)}{\partial B} \quad \text{[S1]}$$

where $\Delta C/C_0$ is the relative capacitance variation and B the magnitude of the external magnetic field. The relative capacitance variation can be represented as:

$$\frac{\Delta C}{C_0} = \frac{C - C_0}{C_0} = \frac{C}{C_0} - 1 \quad \text{[S2]}$$

where C_0 is the initial capacitance without applying the magnetic field and C the resultant capacitance under the external magnetic field. The capacitance is normally defined by:

$$C = \frac{\varepsilon A}{d} \quad \text{[S3]}$$

where ε is the dielectric constant, d the spacing between the facing electrodes and A the facing area of the electrodes (constant in this work). For two-phase composite materials, the dielectric constant can be expressed by [S1, S2]:

$$\varepsilon = \varepsilon_a f_a + \varepsilon_b f_b \quad \text{[S4]}$$

where ε_a and ε_b are the intrinsic dielectric constant of the air component and NdFeB/PDMS component in this case, respectively, and f_a and f_b are the corresponding volume fraction. The volume of t-FMA$_b$@MPS component (V_b) maintains unchanged during the deformation of micro pillars, thus we have

$$f_b = \frac{V_b}{V} = \frac{V_b}{A \cdot d},\ f_a = \frac{V - V_b}{V} = \frac{A \cdot d - V_b}{A \cdot d} \quad \text{[S5]}$$

where $V = A \cdot d$ is the total volume between the two facing electrodes. Then the initial and resultant dielectric constant can be expressed as:

$$\varepsilon_0 = \varepsilon_a f_{a_0} + \varepsilon_b f_{b_0} = \frac{\varepsilon_a A d_0 + (\varepsilon_b - \varepsilon_a) V_b}{A d_0},\ \varepsilon = \varepsilon_a f_a + \varepsilon_b f_b = \frac{\varepsilon_a A d + (\varepsilon_b - \varepsilon_a) V_b}{A d} \quad \text{[S6]}$$

where d_0 is the initial distance between the facing electrodes (constant in this work). With the combination of Equation S2, S3 and S6, we have

$$\frac{\Delta C}{C_0} = \frac{C}{C_0} - 1 = \frac{\varepsilon A/d}{\varepsilon_0 A/d_0} - 1 = \frac{\varepsilon_a A d + (\varepsilon_b - \varepsilon_a) V_b}{\varepsilon_a A d_0 + (\varepsilon_b - \varepsilon_a) V_b} \cdot \frac{d_0^2}{d^2} - 1 \quad \text{[S7]}$$

Thus, the deformation capability of t-FMA$_b$ (i.e., variation in d) induced by external magnetic field mainly contributes to the variation in capacitance, and typically a higher deformation capability (whether deforming downwards or upwards) renders a greater sensitivity as discussed in Fig. 3b, c.

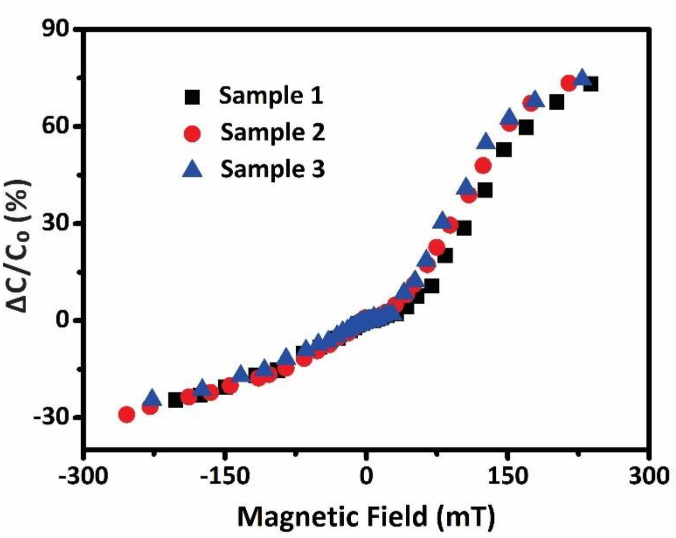

Fig. S13 Relative capacitance variation of the proposed sensor across different samples

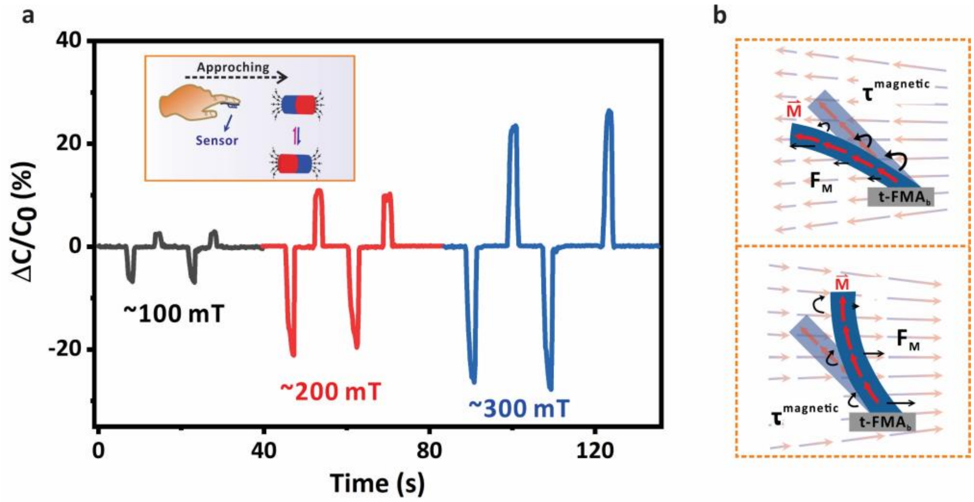

Fig. S14 a) Real-time response of the optimized sensor to different in-plane magnetic fields (~100, 200 and 300 mT). **b**) Mechanism of the bending actuation of t-FMA$_b$@MPA under positive and negative in-plane magnetic fields

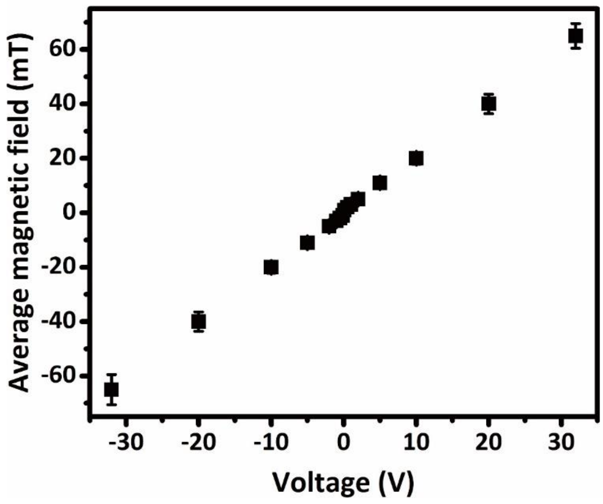

Fig. S15 Surface magnetic field magnitude of the electromagnet (diameter of 50 mm) under operating voltages provided by a power supply. The error bars are standard deviations obtained from at least 5 independent measurements.

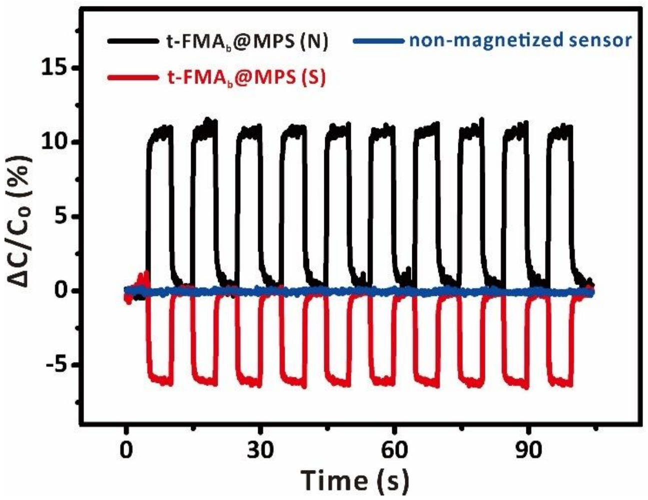

Fig. S16 Real-time response of the sensor based on magnetized and non-magnetized t-FMA$_b$@MPS under positive and negative magnetic fields (with a constant magnitude of 65mT)

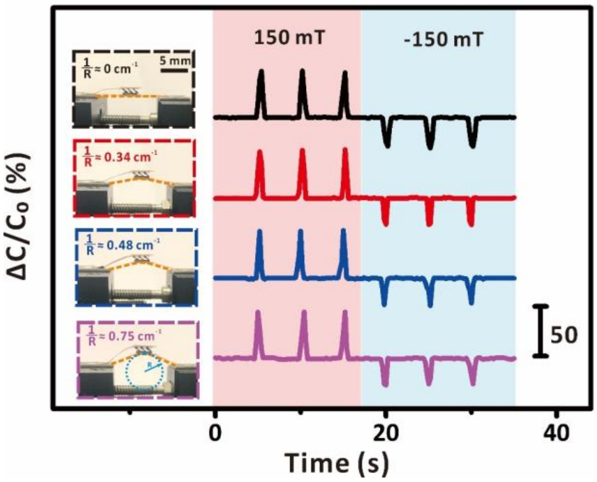

Fig. S17 Real-time electric response of the sensor to both positive and negative magnetic fields (with a constant magnitude of 150 mT) under different bending curvatures ($\frac{1}{R}$ = 0, 0.34, 0.48 and 0.75 cm^{-1}, R is defined as the radius of curvature)

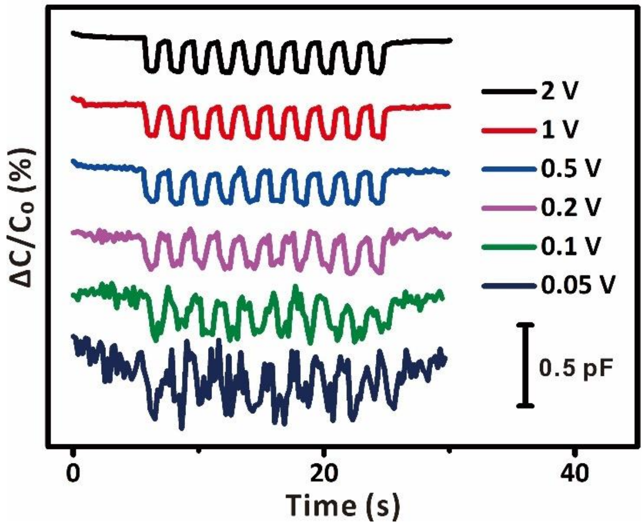

Fig. S18 Real-time electric response of the sensor to the negative magnetic field (with a constant magnitude of 65 mT) with different operating voltages

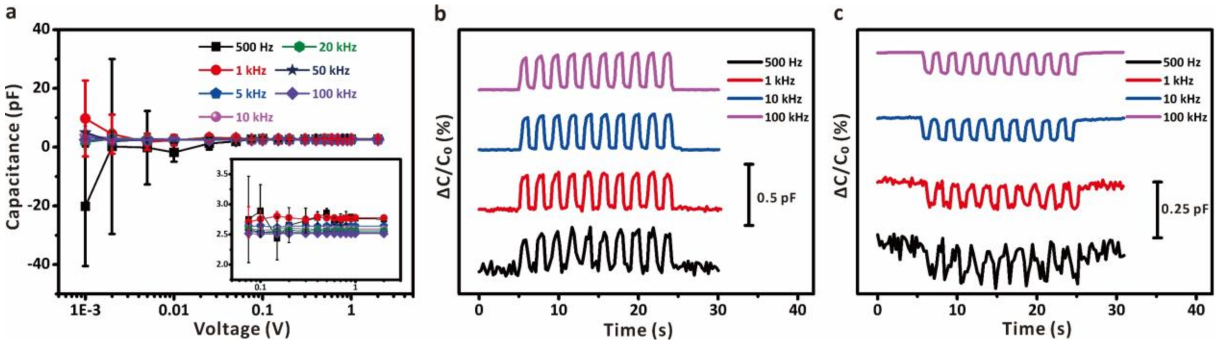

Fig. S19 a) The relationship between the capacitance and operating voltage of the sensor at initial stage under different operating frequencies (0.5, 1, 5, 10, 20, 50 and 100 kHz). The error bars represent the standard deviations of at least ten individual measurements. **b-c**) Real-time electric response of the sensor to the (**b**) positive and (**c**) negative magnetic field with a constant magnitude of 65 mT under a constant operating voltage of 1 V but different operating frequencies. The results reveal that the electric response of the sensor can be well repeated with the operating voltage over 50 mV and the operating frequency over 1kHz.

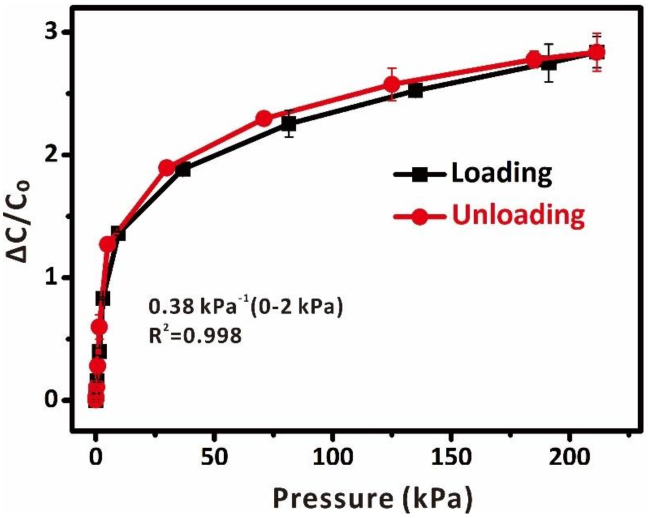

Fig. S20 Pressure test of the capacitive sensor based on t-FMA$_b$@MPS under the dynamic loading/unloading of pressures up to 200 kPa. The error bars are standard deviations obtained from at least five individual measurements.

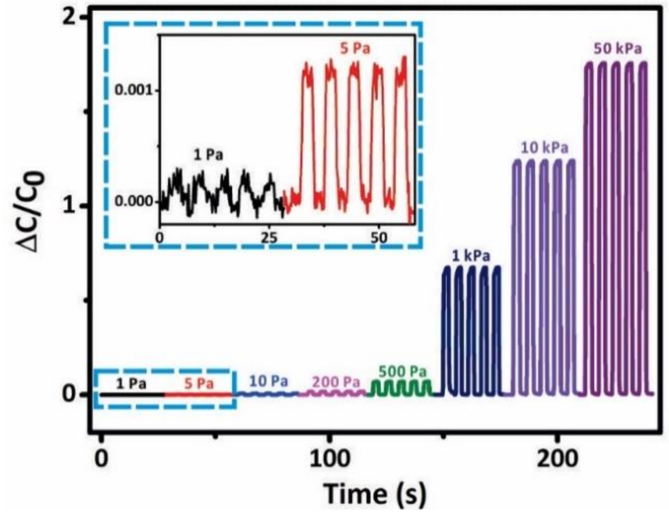

Fig. S21 Stability of the sensor under different pressures. The sensor exhibits high pressure resolution and can repeatably detect an ultra-low pressure of 1 Pa.

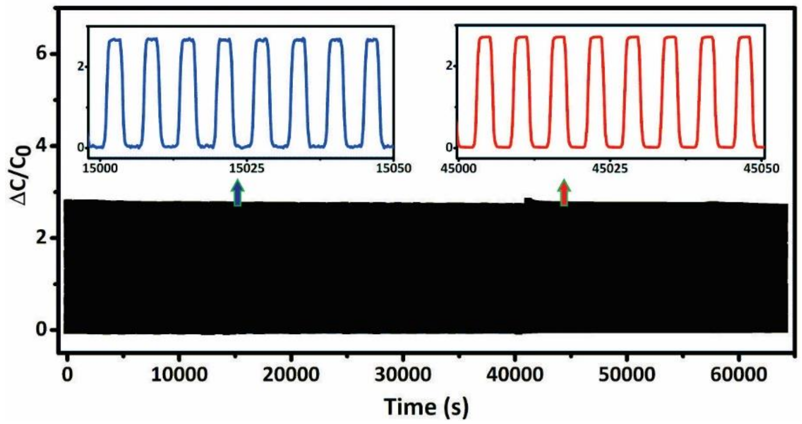

Fig. S22 Long-term stability of the sensor under a periodically loading/unloading of a high pressure of 200 kPa for over 5000 cycles

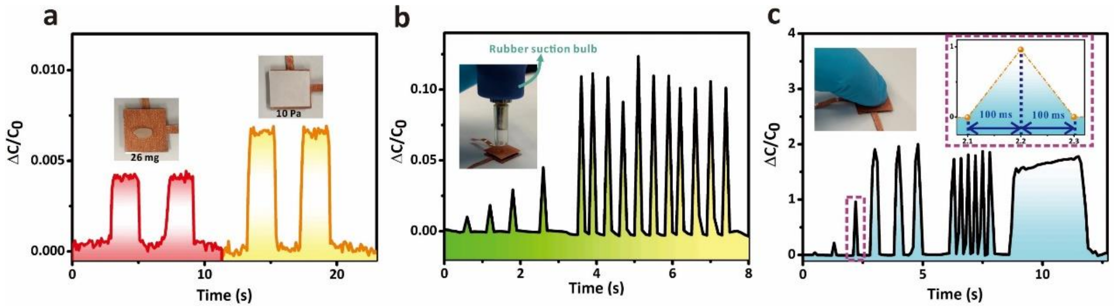

Fig. S23 Real time response of the sensor to repeated ultra-lightweight substance (rice and paper), air flows and finger press. The proposed sensor exhibits fast response/relaxation time of ~100 ms due to the excellent elasticity of PDMS, shown in the inset of Fig. S23c.

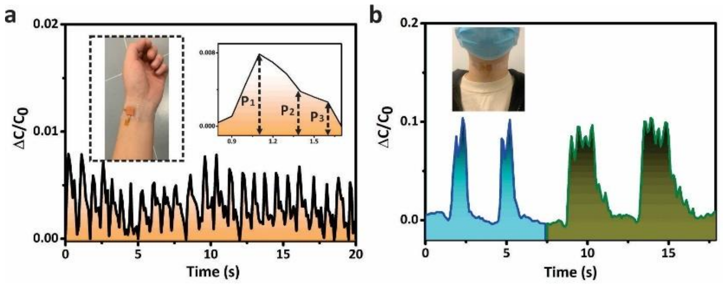

Fig. S24 Wearable applications of the sensor for pulse sensing (**a**) and voice recognition (**b**)

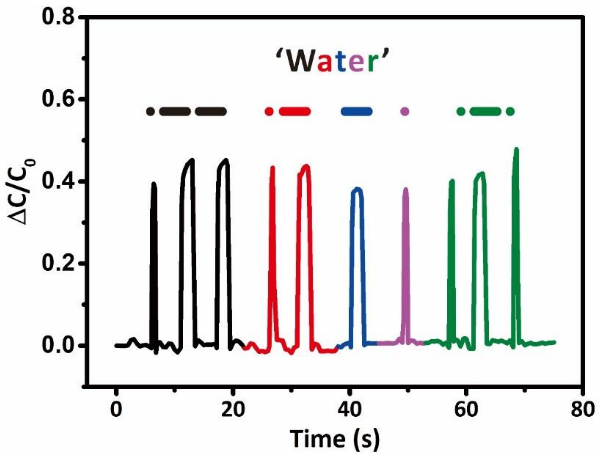

Fig. S25 Real-time response of the sensor to finger press for generating the Morse code message of 'water'. The duration of the pressure was applied for representing the 'dot' and 'dash' symbols in Morse code system.

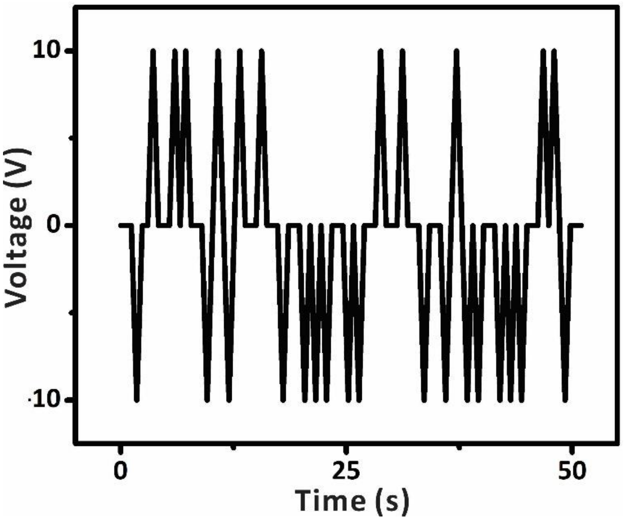

Fig. S26 Input voltage sequence to the electromagnet for generating the magnetic fields encoded with Morse codes of ‘Nice to meet you’. The designed voltage inputs are generated by a digital function generator.

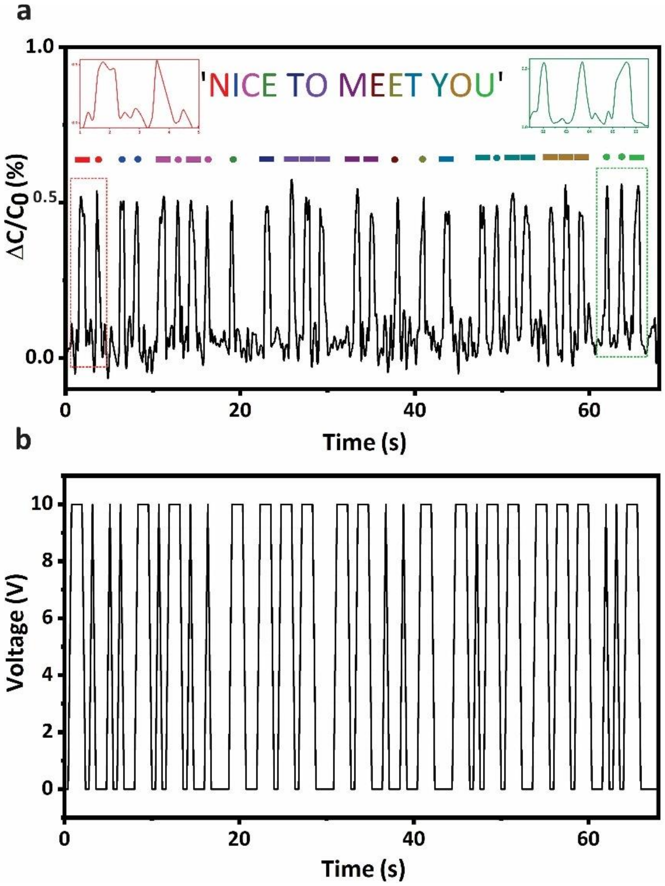

Fig. S27 a) Real-time response of the sensor to electromagnet-induced magnetic fields that carried the Morse code message of ‘nice to meet you’. The duration of magnetic fields was applied for representing the ‘dot’ and ‘dash’ symbols in Morse code system, as revealed by the insets. **b**) Waveform of the voltage supply for the electromagnet to produce the magnetic field for Morse code delivery. In this case, the duration of voltage supply was altered to represent the ‘dot’ and ‘dash’, which finally led to a longer duration to complete the formation of the message when compared with the result in Fig. 4c. Here, the short and long duration to provide the voltage supply for magnetic field generation was 0.4 s and 1.2 s, respectively.

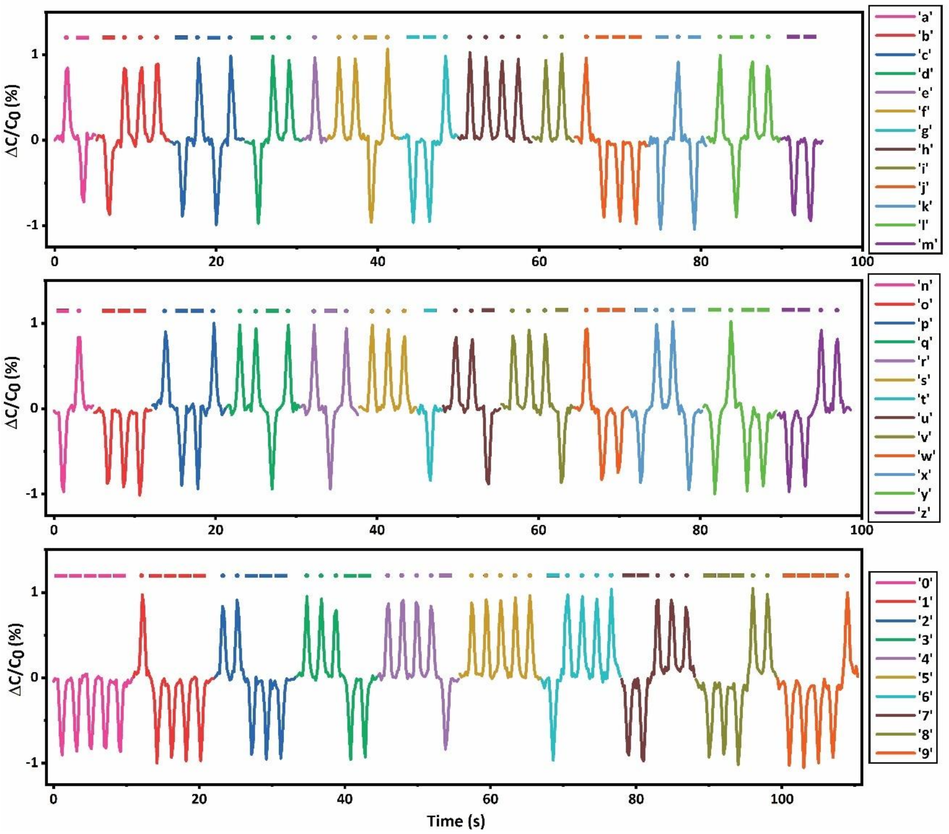

Fig. S28 Real-time capacitance signals and decoded Morse code information of letters and numbers received by the sensor. The positive and negative peaks represent the dots and dashes in Morse code, respectively.

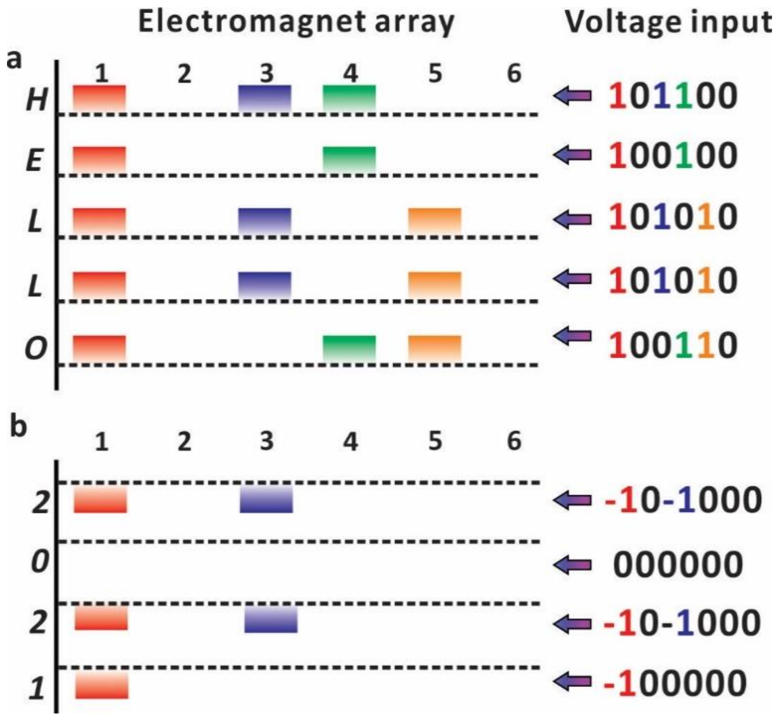

Fig. S29 Real-time voltage input (± 12 V) to the electromagnet array for outputting the magnetic fields encoded with Braille phrases of 'Hello 2021'. The square wave voltage with a period of 2 s was employed to output electromagnetic field for the dot-pattern of Braille. The codes '1' and '-1' here are defined as the voltage inputs of 12 and -12 V, respectively.

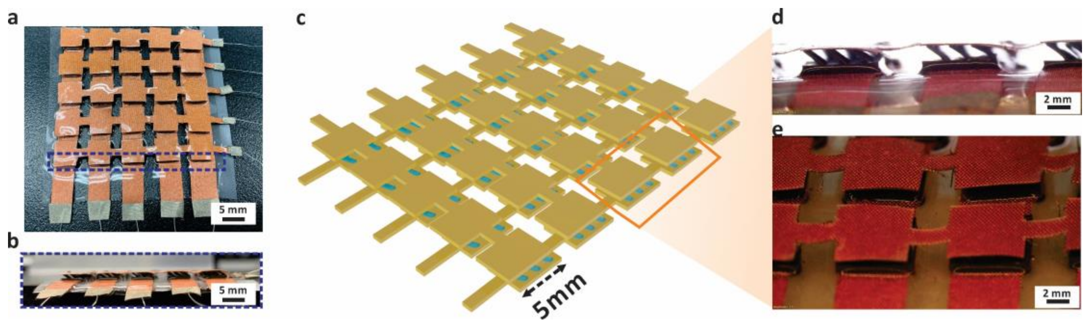

Fig. S30 Photographs (**a** and **b**), schematic figure (**c**) and optical images (**d** and **e**) of 5 × 5 sensor arrays

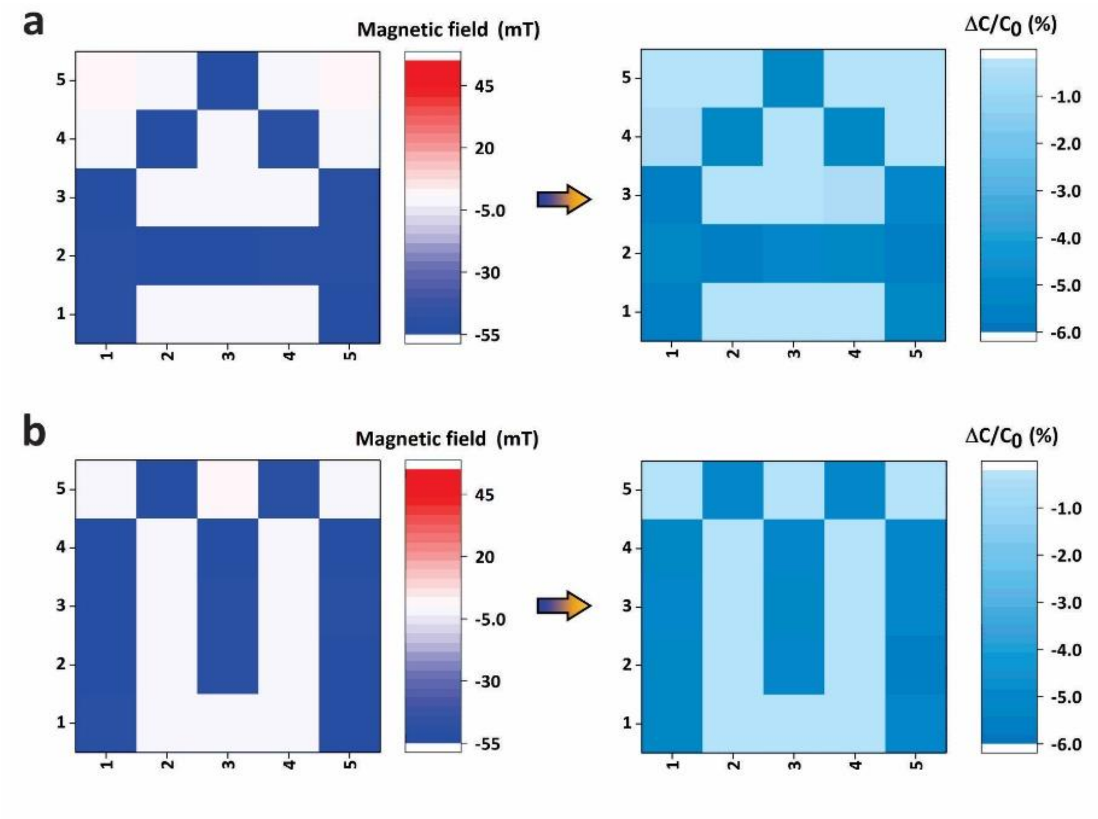

Fig. S31 a-b) Magnetic field mapping of the flexible magnet array that encoded the cryptic letters of 'A' and 'M' (left column) and the capacitance signal mapping perceived by the sensor array (right column)

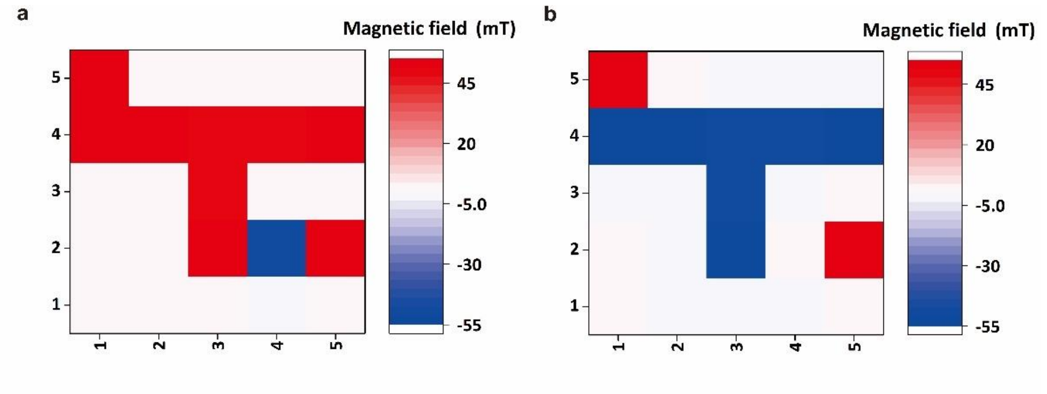

Fig. S32 a-b) Magnetic field mapping of flexible magnet array that encoded the cryptic ID information shown in Fig. 5e (**a**) and 5f (**b**)

Table S1 Key performance comparison of flexible and wearable magnetism sensor

Materials	Type (Measurement scheme)	Sensitivity (Linear range)	Pole distinguishability	Methodology	Refs.
Graphene-coated magnetic cilia array	Resistive	0.3 T^{-1} (0-100mT) 3.5 T^{-1} (100-120 mT) 7.5 T^{-1} (120-160 mT) 12.08 T^{-1} (150-160 mT)	No	In situ growth	[S3]
Carbon aerogel/Iron Rubber	Resistive	16 T^{-1}(0-50 mT)	No	High-temperature carbonization	[S4]
3D PDMS-AgNWs-magnetowhisker	Resistive	1.52 T^{-1} (31.3-40.6 mT)	No	Laser cutting	[S5]
Magnetorheological plastomers/$CuSO_4$/PU	Self-powered based on Electrochemical Reaction	0.05 $V{\cdot}T^{-1}$ (0-106 mT) 0.16 $V{\cdot}T^{-1}$(106-252 mT)	No	Wet etching; magnetron sputter deposition	[S6]
Micro-pyramid NdFeB/PDMS	GMR effect (DC)	-	No	Magnetron sputter deposition; thermal evaporation	[S7]
Flexible organic tribotronic transistor (FOTT)	Triboelectric	1.6 T^{-1} (1-150 mT)	No	Magnetron sputter deposition	[S8]
PET/ITO/[ZnS:Cu/PVDF-TrFE-CFE]/Fe_3O_4-MWNTs	Magneto-interactive Electroluminescence	248.98 T^{-1} (70-210 mT)	No	Magnetron sputter deposition	[S9]
$Ni_{80}Fe_{20}$/Kapton	GMI	12 $k\Omega{\cdot}T^{-1}$(0.25-0.8 mT)	No	Lithography and electron beam	[S10]
Bismuth/PI	Hall effect (DC@ 50 mA)	2.3 $V{\cdot}(A \cdot T)^{-1}$(0-400 mT)	Yes	Lithography and Lift-off technology	[S11]
$Fe_{81}Ni_{19}$/PET	AMR (DC@1 mA)	5.4 T^{-1}(5 nT- 750 µT)	No	Photolithography	[S12]
$Ni_{80}Fe_{20}$/PET	Planar Hall effect (DC@ 5 mA)	0.86 V/T (20 nT- 3 mT)	Yes	Lithography and electron beam	[S13]
Magnetized tilted micropillar array based on NdFeB/PDMS	Capacitive (AC)	1.3 T^{-1} (1-20 mT) and 4.5 T^{-1} (20-200 mT, N pole); 1.4 T^{-1} (1-220 mT, S pole)	Yes	Micro-engraving technology	**Our work**

Section S1 Establishment of COMSOL Model

The models were established in a two-dimensional structure using COMSOL Multiphysics software. For simplification, a tilted micro pillar (300 µm in diameter, 2 mm in length and 45°

in tilted angle) and a substrate (2 mm in length and width, and 500 μm in thickness) were employed as the geometric configuration to simulate the distribution of magnetic field of the tilted flexible micromagnet. PDMS with the relative permeability of 4000 and 1 was set as the materials of the magnetized tilted micro pillar and substrate, respectively. The geometric configurations were surrounded by an air domain with diameter of 30 mm. The mesh was controlled by the physics interfaces with extra fine sizes. The Magnetic Fields (no Currents) module was employed to trace the magnetic scalar potential and magnetic flux density, with the governing equations $\boldsymbol{H} = -\nabla V_m$ and $\nabla \cdot \boldsymbol{B} = 0$, where $\boldsymbol{H}$ is the magnetic field vector, $\boldsymbol{B} = \mu_0 \mu_r \boldsymbol{H}$ is the magnetic flux density vector and V_m the magnetic scalar potential. The boundary conditions were set as $\boldsymbol{n} \cdot \boldsymbol{B} = 0$. The initial value of magnetic scalar potential was set as 0. The constitutive relation between the magnetic field and magnetization was governed by $\boldsymbol{B} = \mu_0(\boldsymbol{H} + \boldsymbol{M})$, where $\boldsymbol{M}$ is the magnetization vector. The y-component of magnetization was set as1 A/m for the vertical magnetization. The x and y components of magnetization were set as 1 A/m to build the tilted magnetization. The magnetic scalar potential and magnetic flux density were calculated to describe the distribution of magnetic field.

Supplementary References

[S1] S.R.A. Ruth, L. Beker, H. Tran, V.R. Feig, N. Matsuhisa et al., Rational design of capacitive pressure sensors based on pyramidal microstructures for specialized monitoring of biosignals. Adv. Funct. Mater. **30**, 1903100 (2019). https://doi.org/10.1002/adfm.201903100

[S2] Y. Wan, Z. Qiu, J. Huang, J. Yang, Q. Wang et al., Natural plant materials as dielectric layer for highly sensitive flexible electronic skin. Small **14**, 1801657 (2018). https://doi.org/10.1002/smll.201801657

[S3] Y.-F. Liu, Y.-F. Fu, Y.-Q. Li, P. Huang, C.-H. Xu et al., Bio-inspired highly flexible dual-mode electronic cilia. J. Mater. Chem. B **6**, 896-902 (2018). https://doi.org/10.1039/C7TB03078A

[S4] P. Huang, D. Tan, Q.-M. Li, Y.-Q. Li, Y.-Q. Fu et al., Dual-mode carbon aerogel/iron rubber sensor. ACS Appl. Mater. Interfaces **12**, 8674-8680 (2020). https://doi.org/10.1021/acsami.9b20662

[S5] L. Ding, Y. Wang, C. Sun, Q. Shu, T. Hu et al., Three-dimensional structured dual-mode flexible sensors for highly sensitive tactile perception and noncontact sensing. ACS Appl. Mater. Interfaces **12**, 20955-20964 (2020). https://doi.org/10.1021/acsami.0c03996

[S6] J. Xu, L. Pei, J. Li, H. Pang, Z. Li et al., Flexible, self-powered, magnetism/pressure dual-mode sensor based on magnetorheological plastomer. Compos. Sci. Technol. **183**, 107820 (2019). https://doi.org/10.1016/j.compscitech.2019.107820

[S7] J. Ge, X. Wang, M. Drack, O. Volkov, M. Liang et al., A bimodal soft electronic skin for tactile and touchless interaction in real time. Nat. Commun. **10**, 4405 (2019). https://doi.org/10.1038/s41467-019-12303-5

[S8] J. Zhao, H. Guo, Y.K. Pang, F. Xi, Z.W. Yang et al., Flexible organic tribotronic transistor for pressure and magnetic sensing. ACS Nano **11**, 11566-11573 (2017). https://doi.org/10.1021/acsnano.7b06480

[S9] S.W. Lee, S. Baek, S.-W. Park, M. Koo, E.H. Kim et al., 3D motion tracking display enabled by magneto-interactive electroluminescence. Nat. Commun. **11**, 6072 (2020). https://doi.org/10.1038/s41467-020-19523-0

[S10] A. Alfadhel, J. Kosel, Magnetic nanocomposite cilia tactile sensor. Adv. Mater. **27**, 7888-7892 (2015). https://doi.org/10.1002/adma.201504015

[S11] M. Melzer, J.I. Mönch, D. Makarov, Y. Zabila, G.S. Cañón Bermúdez et al., Wearable magnetic field sensors for flexible electronics. Adv. Mater. **27**, 1274-1280 (2015). https://doi.org/10.1002/adma.201405027

[S12] G.S. Cañón Bermúdez, H. Fuchs, L. Bischoff, J. Fassbender et al., Electronic-skin compasses for geomagnetic field-driven artificial magnetoreception and interactive electronics. Nat. Electron. **1**, 589–595 (2018). https://doi.org/10.1038/s41928-018-0161-6

[S13] P.N. Granell, G. Wang, G.S. Cañon Bermudez, T. Kosub, F. Golmar et al., Highly compliant planar Hall effect sensor with sub 200 nT sensitivity. npj Flex. Electron. **3**, 3 (2019). https://doi.org/10.1038/s41528-018-0046-9
